# The extracellular matrix proteoglycan fibromodulin is upregulated in clinical and experimental heart failure and affects cardiac remodeling

**DOI:** 10.1371/journal.pone.0201422

**Published:** 2018-07-27

**Authors:** Kine Andenæs, Ida G. Lunde, Naiyereh Mohammadzadeh, Christen P. Dahl, Jan Magnus Aronsen, Mari E. Strand, Sheryl Palmero, Ivar Sjaastad, Geir Christensen, Kristin V. T. Engebretsen, Theis Tønnessen

**Affiliations:** 1 Institute for Experimental Medical Research, Oslo University Hospital and University of Oslo, Oslo, Norway; 2 KG Jebsen Cardiac Research Center and Center for Heart Failure Research, University of Oslo, Oslo, Norway; 3 Centre for Molecular Medicine Norway, Oslo University Hospital and University of Oslo, Oslo, Norway; 4 Research Institute of Internal Medicine, Oslo University Hospital, Oslo, Norway; 5 Department of Cardiology, Oslo University Hospital, Oslo, Norway; 6 Bjørknes College, Oslo, Norway; 7 Department of Surgery, Vestre Viken Hospital, Drammen, Norway; 8 Department of Cardiothoracic Surgery, Oslo University Hospital, Oslo, Norway; Scuola Superiore Sant'Anna, ITALY

## Abstract

Pressure overload of the heart leads to cardiac remodeling that may progress into heart failure, a common, morbid and mortal condition. Increased mechanistic insight into remodeling is instrumental for development of novel heart failure treatment. Cardiac remodeling comprises cardiomyocyte hypertrophic growth, extracellular matrix alterations including fibrosis, and inflammation. Fibromodulin is a small leucine-rich proteoglycan that regulates collagen fibrillogenesis. Fibromodulin is expressed in the cardiac extracellular matrix, however its role in the heart remains largely unknown. We investigated fibromodulin levels in myocardial biopsies from heart failure patients and mice, subjected fibromodulin knock-out (FMOD-KO) mice to pressure overload by aortic banding, and overexpressed fibromodulin in cultured cardiomyocytes and cardiac fibroblasts using adenovirus. Fibromodulin was 3-10-fold upregulated in hearts of heart failure patients and mice. Both cardiomyocytes and cardiac fibroblasts expressed fibromodulin, and its expression was increased by pro-inflammatory stimuli. Without stress, FMOD-KO mice showed no cardiac phenotype. Upon aortic banding, left ventricles of FMOD-KO mice developed mildly exacerbated hypertrophic remodeling compared to wild-type mice, with increased cardiomyocyte size and altered infiltration of leukocytes. There were no differences in mortality, left ventricle dilatation, dysfunction or expression of heart failure markers. Although collagen amount and cross-linking were comparable in FMOD-KO and wild-type, overexpression of fibromodulin in cardiac fibroblasts *in vitro* decreased their migratory capacity and expression of fibrosis-associated molecules, i.e. the collagen-cross linking enzyme lysyl oxidase, transglutaminase 2 and periostin. In conclusion, despite a robust fibromodulin upregulation in clinical and experimental heart failure, FMOD-KO mice showed a relatively mild hypertrophic phenotype. In cultured cardiac fibroblasts, fibromodulin has anti-fibrotic effects.

## Introduction

Pressure overload of the heart, as seen in patients with hypertension or aortic stenosis, leads to cardiac remodeling and may progress into heart failure (HF) [1]. HF carries high morbidity, mortality and societal costs, affecting millions of patients worldwide [1], suggesting that current therapy does not adequately target the underlying mechanisms [2]. Increased mechanistic insight into cardiac remodeling and HF will likely contribute to development of novel HF treatment.

Cardiac remodeling encompasses cardiomyocyte (CM) hypertrophic growth or death, and extracellular matrix (ECM) alterations such as increased levels of fibrillar collagens (i.e. fibrosis) and collagen cross-linking, fibroblast-to-myofibroblast transdifferentiation, and inflammation [3]. The ECM is increasingly recognized as an important mediator of cardiac remodeling [4–8]. Proteoglycans are glycosylated proteins, consisting of a core protein with covalently attached glycosaminoglycan (GAG) chains [9]. Most proteoglycans are localized to, or extending into the ECM, and they are increasingly believed to play a role in cardiac remodeling [4, 5, 10–12].

Small leucine-rich proteoglycans (SLRPs) are low molecular weight proteoglycans localized to the ECM, and constitute the largest family of proteoglycans with 18 members [9, 13]. They are characterized by their leucine-rich repeats (LRRs) and are expressed in most ECMs, including the cardiac ECM. SLRPs bind collagens, including the fibrillar collagens in the heart (collagen types I and III), regulate collagen fibrillogenesis, modulate signaling and affect cell growth [9, 10, 13]. Although the exploration of roles of SLRPs in cardiac remodeling are at its beginning, lumican (LUM), biglycan (BGN), decorin (DCN) and osteoglycin (OGN) are believed to be important in ECM, regulating collagen amount and fibrillogenesis in the heart [5, 10].

Fibromodulin (FMOD) is a collagen-binding keratan sulfate SLRP expressed in connective tissues and cartilage, binding to collagen, regulating collagen fibrillogenesis and influencing on collagen cross-linking [13–15]. FMOD expression is increased in inflammatory, fibrotic and wound healing processes in liver, kidney, lung and skin [16–19]. In skin scars, FMOD regulates fibrotic responses through interaction with transforming growth factor (TGF)β [20, 21]. Although FMOD is expressed in the heart [22–25], its role remains largely unknown. Here, we investigated the role of FMOD in cardiac remodeling responses in myocardial biopsies from HF patients, *in vivo* by subjecting FMOD knock-out (FMOD-KO) mice to aortic banding (AB), and *in vitro* by adenoviral overexpression of FMOD in CM and cardiac fibroblast (CFB) primary cultures.

## Materials and methods

### Ethics

The human biopsy protocol was reviewed and approved by the Regional Committee for Medical Research Ethics (REK ID 07482a), the South-Eastern Regional Health Authority, Norway, and was in accordance with the Declaration of Helsinki. Written informed consent was signed by all patients and by the next of kin of controls. Animal experiments were reviewed and approved by the Norwegian National Animal Research Committee (protocol IDs 7018 and 3170). The NIH Guide for the Care and Use of Laboratory Animals was followed (NIH publication no. 85–23, revised 2011).

### Myocardial biopsies from patients

Patients were treated according to hospital guidelines. Left ventricle (LV) tissue biopsies were collected from beating hearts immediately after explantation from patients with end-stage, dilated HF with reduced ejection fraction (HFrEF) undergoing cardiac transplantation. LV tissue biopsies from non-diseased hearts, considered but found unsuitable for transplantation, were sampled as controls. Tissue biopsies were snap-frozen in liquid nitrogen and stored at -80°C.

### Mouse lines

A wild-type (WT) mouse cohort on C57BL/6J background (BomTac, Taconic, Skensved, Denmark, hereafter referred to as the WT cohort) was subjected to pressure overload for 18 weeks (w) to characterize myocardial expression of FMOD over time. A cohort of FMOD-KO mice [15] on C57BL/6N background were kindly provided by Professor Åke Oldberg, Lund University, Lund, Sweden. FMOD-KO mice were backcrossed onto C57BL/6N background and background was confirmed satisfactory (>98.7%) by the Charles River Laboratories (Wilmington, MA). Heterozygous FMOD-KO intercrosses provided WT and FMOD-KO littermates, which were used for homozygous breeding of the FMOD-KO mice and WT controls for comparison. Genotyping was performed from ear biopsies using primers as described before [15].

### Experimental mice model of cardiac pressure overload

Mice were randomly assigned to sham or AB surgery at 7-9w of age and subjected to LV pressure overload by banding of the ascending aorta, as previously described [26]. Sham-operated animals underwent the same procedure without tightening of the suture around the aorta. Briefly, mice were intubated and ventilated with 2% isoflurane and 98% oxygen. The thoracic cavity was accessed through an incision in the left second intercostal space and in AB mice an 8–0 silk ligature was tied around the ascending aorta and a 26G blunted needle to standardize the degree of constriction. Animals received post-operative analgesia by subcutaneous injection of 0.02 mL buprenorphine (0.3 mg/mL). Surgery was performed by an experienced mouse surgeon blinded to genotype.

### Mouse blood pressure measurements

Blood pressure was measured by an experienced researcher blinded to genotype, using the non-invasive the CODA tail-cuff system according to protocol (Kent Scientific Corporation, Torrington, CT).

### Mouse echocardiography

Echocardiography of mice breathing 1.75% isoflurane on a mask was performed using the VEVO 2100 system (VisualSonics, Toronto, Canada), by an experienced researcher blinded to genotype and type of surgery. Animals with sufficient degree of aortic constriction (maximal flow velocity (Vmax) of 3.5–4.5 m/s over the stenosis) 24h after AB were included (Mean Vmax at 24h were 3.95 (WT) and 3.99 (FMOD-KO)). From a total of n = 101 AB mice, 9 mice were excluded due to insufficient degree of stenosis. The WT cohort was analyzed by echocardiography at 24h, 1, 3, 16 and 18w post-surgery to assess cardiac dimensions and function. From the FMOD-KO and respective WT cohort of mice echocardiography was performed at 2, 4, 6, 8, 10 and 12w post-surgery to assess cardiac dimensions and function. LV posterior wall thickness in diastole (LVPWd), interventricular septal thickness in diastole (IVSd), LV internal diameter in diastole (LVIDd) and systole (LVIDs) were obtained from M-mode recordings. Fractional shortening (FS) was calculated from M-mode echocardiography images, FS = 100× ((LVIDd-LVIDs)/LVIDd). LV ejection fraction (EF) was calculated by the cube method, EF = 100× ((LVIDd^3^-LVIDs^3^)/LVIDd^3^).

### Mouse tissue harvesting

Mice were sacrificed by cervical dislocation under deep anesthesia. Tissue from the WT cohort was harvested 24h, 1, 3, 16 and 18w post-surgery, and tissue from C57BL/6N FMOD-KO and WT controls were harvested at 2, 4 or 12w post-surgery. Hearts and lungs were rapidly excised, rinsed in 1X phosphate-buffered saline (PBS), blotted dry, weighed, the LV dissected, snap-frozen in liquid nitrogen, and stored at -80°C for molecular analyses. For histology, the heart was sectioned at the midventricular short axis plane and the basal fixed in 4% formaldehyde. The apical part of the LV was dissected and snap-frozen in liquid nitrogen for molecular analyses. Body weight, heart weight, lung weight, and tibia length were measured at sacrifice.

### Histology

Formaldehyde-fixed heart tissue was paraffin-embedded and sectioned. Midventricular sections (4 μm) were stained following manufactures protocols. Staining with Wheat Germ Agglutinin (WGA) was performed to measure CM cross sectional area (CSA). High-resolution images of histological sections were acquired using an automated slide scanner system (Axio Scan Z1, Carl Zeiss Microscopy, Munich, Germany). Images were inspected using the Zen Lite Blue software (Carl Zeiss Microscopy). CSA was analyzed and calculated blinded to genotype using ImageJ (NIH) software. Staining with Picrosirius Red was performed to measure collagen cross-linking under polarized light [27]. Images of histological sections were acquired using a confocal laser scanning microscope (LSM800, Carl Zeiss Microscopy, Munich, Germany). Analysis and calculations were performed blinded to genotype using ImageJ (NIH). Amount of collagen cross-linking was quantified by measuring area of cross-linking divided by the area of the whole midventricular section (%).

### High performance liquid chromatography

Collagen content in LV from FMOD-KO and WT mice was assessed as hydroxyproline incorporation using HPLC and the AccQ-Fluor reagent kit (Waters Corporation Milford, MA), as described previously [26]. Briefly, the whole LV was pulverized in liquid nitrogen, 15 mg hydrolyzed in 6 M HCl over night at 112°C, dried under vacuum, and dissolved in AccQ-Fluor borate buffer. HPLC was performed using the HPLC column and the Ultimate 3000 (Nerliens Meszansky, Oslo, Norway). Hydroxyproline was detected by fluorescence following excitation at 250 nm and recorded at emission at 395 nm, using hydroxyproline as standard (Fluka, Buchs SG, Switzerland).

### Primary cultures of neonatal cardiac myocytes and fibroblasts

Primary ventricular cells were isolated from Wistar rats 1–3 days of age, i.e. neonatal CFB and CM, as described [28]. Briefly, hearts were excised and trimmed for atrial tissue prior to mechanical digestion in a collagenase solution. Cells were transferred to uncoated culture flasks with serum-containing medium for 20 min, allowing CFB to attach. The unattached CM fraction was seeded onto gelatin/fibronectin-coated six-well plates overnight in serum-containing medium at a density of 3.75 x 10^5^ cells/ml. CFB were cultured in serum-containing medium for up to one week, passaged and seeded onto six-well plates at a density of 1.8 x 10^5^ cells/ml. Cells were kept in a 37°C, 5% CO_2_ humidified incubator. The purity of similar CFB and CM cultures has been confirmed previously by an 800-fold higher expression of cardiac troponin I (TNNI) in CM vs. CFB [28].

CFB and CM were serum starved for 24h prior to treatment for 24h with lipopolysaccharide (LPS, 1μg/ml) or the NFκB-inhibitor SM7368 (10μM), alone or in combination, washed twice in PBS and harvested. Non-treated cells followed the same protocol, except treatment, and were used as controls. Viral transduction of CFB and CM was performed using adenovirus serotype 5 encoding human FMOD (AdFMOD, #ADV-209185, Vector Biolabs, Malvern, PA) or empty vector control (AdVeh, #1300, Vector Biolabs) in serum-containing medium for 24h, as previously described [29]. Virus titers in CM and CFB were 2.5 x 10^7^ and 5 x 10^7^ PFU/ml, respectively. After 24h cells were washed in PBS and serum-free medium was added. Medium, RNA, or protein were harvested from the cells 72h after viral transduction. Non-treated cells followed the same protocol, except transduction, and also served as controls. Experiments were conducted in separate cell culture isolations (i.e. 2–5 biological replicates), and for each isolation we had 2–10 plate well replicates (i.e. technical replicates). The numbers of replicates (n) given in figure legends are the sum of biological replicates × technical replicates.

### Cardiomyocyte protein synthesis assay

The radioactive [^3^H] leucine incorporation protein synthesis assay was performed and quantified essentially as described [29]. In brief, CM were transduced with AdFMOD or AdVeh for 24h prior to culturing in serum-free medium containing 1.25 μCi/ml [^3^H] leucine (Perkin Elmer, Waltham, MA) for 48h. At harvest, cells were washed in 95% ethanol and lysed in 0.2M NaOH. Lysates were diluted in Pico-Fluor 40 (PerkinElmer) and [^3^H] leucine incorporation quantified as counts per min (CPM) using the Wallac Winspectral 1414 liquid scintillation counter (PerkinElmer). Samples were measured in duplicates and serum treated cells were used as positive control. Experiments were conducted in three separate cell culture isolations (i.e. three biological replicates), and with 3–12 technical replicates for each isolation.

### Cardiac fibroblast scratch assay

A scratch assay was performed, as described [29], to study the migration of CFB in presence or absence of FMOD. CFB were seeded into 12-well plates at a density of 1 × 10^5^ cells/ml and transduced with AdVeh or AdFMOD for 24h prior to serum starvation for 24h. Non-transduced cells were used as controls. The cell monolayer was scratched with a sterile plastic pipette tip to create a gap. The culture medium was replaced with fresh media immediately after scratching. Samples with a gap of 1.6mm ± 0.1mm were compared. Images were taken at 0, 6, 12, 18 and 24h following scratching using an Eclipse Ts100 phase contrast microscope (Nikon, Tokyo, Japan), the cell-free area was measured in ImageJ (NIH), and normalized to the cell-free area measured at the time of scratch (time 0) (%).

### HEK293 cell cultures

Human endothelial kidney (HEK) 293 cells were cultured according to supplier protocols, and transfected with a pcDNA3.1 plasmid encoding human FMOD (NP_002014.2), FMOD-His or FMODΔGAG (custom made, Genscript Corporation) using Lipofectamine 2000 (Invitrogen, Paisley, UK), as described [28, 29]. FMODΔGAG was made by site-directed mutagenesis of GAG N-glycosylation attachment sites in FMOD: Asn (N) for Ala (A) residues, N127A, N166A, N201A, N291A, N341A. Cells non-transfected or transfected with empty pcDNA3.1 served as controls. Medium from transfected HEK293 cells was cleared by centrifugation at 5000 g and frozen. RNA or protein were harvested from cells 24h after transfection and stored at -70°C.

### RNA extraction and quantitative real-time PCR

RNA was extracted from cells and LV tissue as described [28–30], using RNeasy mini (Qiagen Nordic, Oslo, Norway). RNA concentration was measured using the Nanodrop ND-1000 Spectrophotometer (Thermo Scientific, Wilmington, DE). RNA quality was determined using the 2100 Bioanalyzer (Agilent Technologies, Santa Clara, CA). cDNA was made using iSCRIPT (BioRad Laboratories, Inc., Hercules, CA) according to manufacturer’s protocol. Pre-designed TaqMan assays (Applied Biosystems, Foster City, CA) were used to determine gene expression (S1 Table). Results were detected in duplicates on a 7900HT Fast Real Time PCR System, and data analyzed using Sequence Detection Software 2.3 (Applied Biosystems). RPL4 or RPL32 were used as reference genes.

### Protein extraction and immunoblotting

Protein was extracted from cells and LV tissue as described [28, 29]. Tissues were homogenized using a Polytron 1200 in a 1X PBS-based lysis buffer containing 1% Triton X-100 (Sigma-Aldrich, St. Louis, MO), 0.1% Tween-20 (Sigma-Aldrich, St. Louis, MO), 0.1% sodium dodecyl sulfate (SDS), protease inhibitors (Complete EDTA-free tablets, Roche Diagnostics, Oslo, Norway) and phosphatase inhibitors (PhosStop, Roche Diagnostics). Cell lysates were harvested using the same lysis buffer. Samples were spun at 20 000 g for 10 min at 4°C and the supernatant stored at -70°C. Harvested medium was cleared by centrifugation at 5000 g for 15 min at 4°C and the supernatant was frozen. Protein concentrations were measured using Micro BCA kit (Thermo Fisher Scientific, Waltham, MA). For lumican immunoblotting, 20 μg protein was deglycosylated using PNGaseF (1 μl/1 hr/37°C), as described [22]. SDS-PAGE and blotting was performed according to the Criterion BIO-RAD protocol. Membranes were blocked in non-fat dry milk (Sigma-Aldrich, St. Louis, MO), casein (Roche Diagnostics, Oslo, Norway) or bovine serum albumin (BSA, Bio-Rad) prior to incubation with primary antibodies (S2 Table) and appropriate HRP-conjugated secondary antibodies. Membranes were developed using ECL Plus Western Blotting Detection System (GE Healthcare, UK) in the Las-4000 (Fujifilm, Tokyo, Japan). Blots were stripped using the Western blot stripping buffer (210591, Thermo Scientific). Images were quantified and processed using ImageJ (NIH) and Adobe Photoshop CC 2017.

### Statistics

Data are given as means ± standard error of mean (SEM). Statistical analyses were performed using GraphPad Prism 7. Statistical significance was accepted for p≤0.05. The statistical tests applied were unpaired t-test, one-way ANOVA with Bonferroni, Dunn`s or Holm-Sidak post-hoc test, and two-way ANOVA, where appropriate. Pearson regression analysis was used for correlations.

## Results

### Fibromodulin is upregulated in clinical heart failure

**Fig 1:**
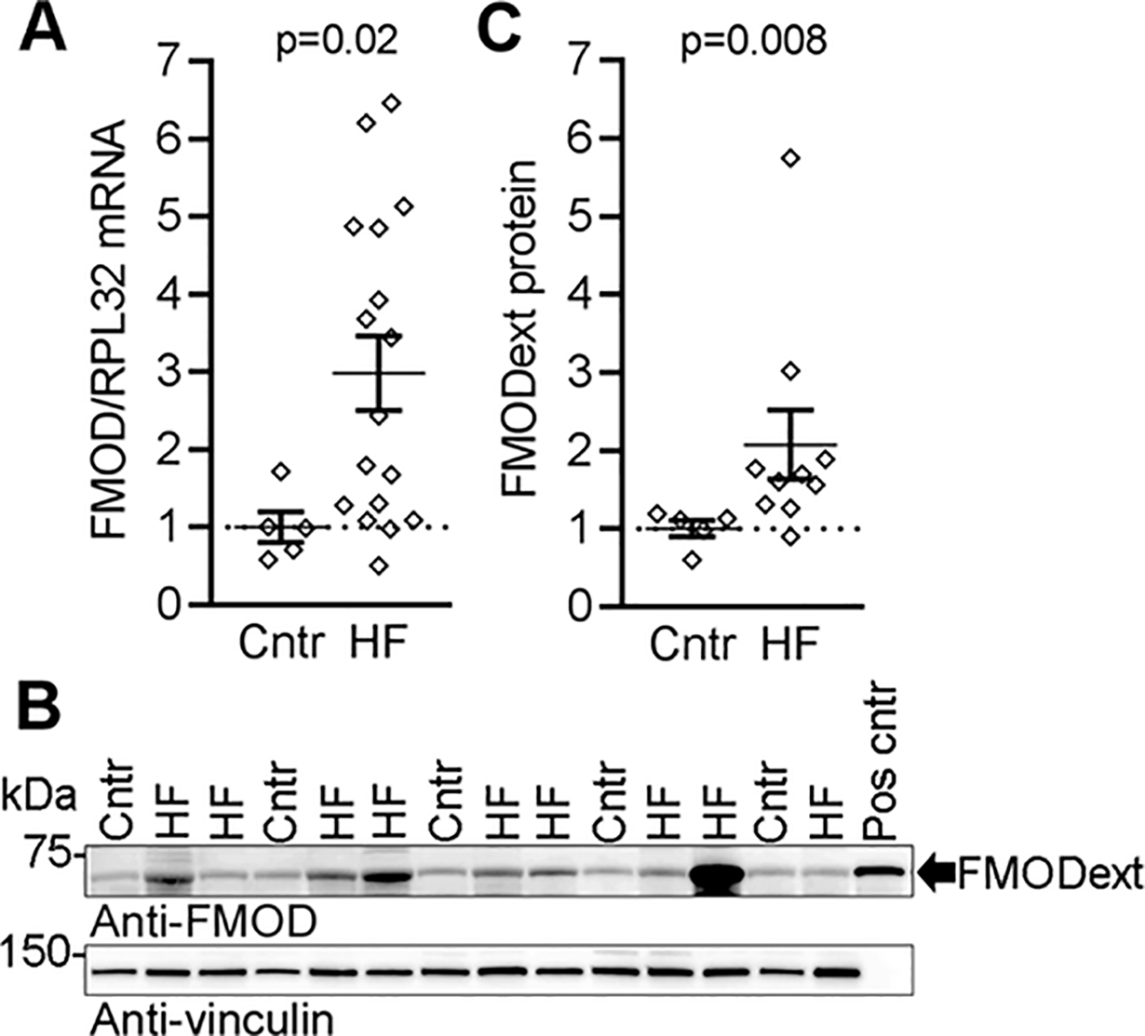
Fibromodulin is upregulated in hearts of patients with heart failure. (A) FMOD mRNA expression in left ventricular biopsies from explanted hearts of end-stage dilated heart failure (HF) patients compared to controls (Cntr), n = 5–17. Ribosomal protein L32 (RPL32) was used as reference gene. (B) Representative immunoblots and (C) quantitative levels of extracellular FMOD (FMODext, ~60kDa) protein in HF patients and Cntr, n = 5–10. FMODext from cell culture medium of FMOD-transfected HEK293 cells was used as positive control (Pos cntr), see S1 Fig for details. Vinculin was used for loading control. HF patient characteristics are found in S3 Table. Data are shown as single data points and mean±SEM. Statistical differences were tested using an unpaired t-test, HF vs. Cntr.

To investigate whether FMOD levels were altered in the failing human myocardium, we assessed FMOD mRNA and protein in LV of explanted hearts of patients with end-stage, dilated HF. The patients had an average LV ejection fraction of 19% and were classified in New York Heart Association classes III-IV (S3 Table). They had elevated circulating levels of pro- brain natriuretic peptide levels, and dilatation was evident from an average LVIDd of 7.5 cm. Importantly, we found a 3-fold up-regulation of FMOD mRNA in LV myocardium from HF patients compared to controls (Fig 1A). FMOD was overexpressed in HEK 293 cells to identify the protein band on immunoblots corresponding to the secreted, extracellular FMOD. FMOD was found in the cell lysate as a ~50 kDa intracellular protein (A-B in S1 Fig). The non-glycosylated FMOD core protein (FMODΔGAG) was found in the cell lysate at the expected size (42 kDa) and was not secreted (B-C in S1 Fig). The FMOD protein was secreted into the cell medium as a ~60 kDa proteoglycan, i.e. the expected size [14] (FMODext, C in S1 Fig). Importantly, in the myocardium of HF patients, the FMODext protein was increased ~2-fold compared to controls (Fig 1B and 1C).

### Fibromodulin is upregulated in experimental heart failure

**Fig 2:**
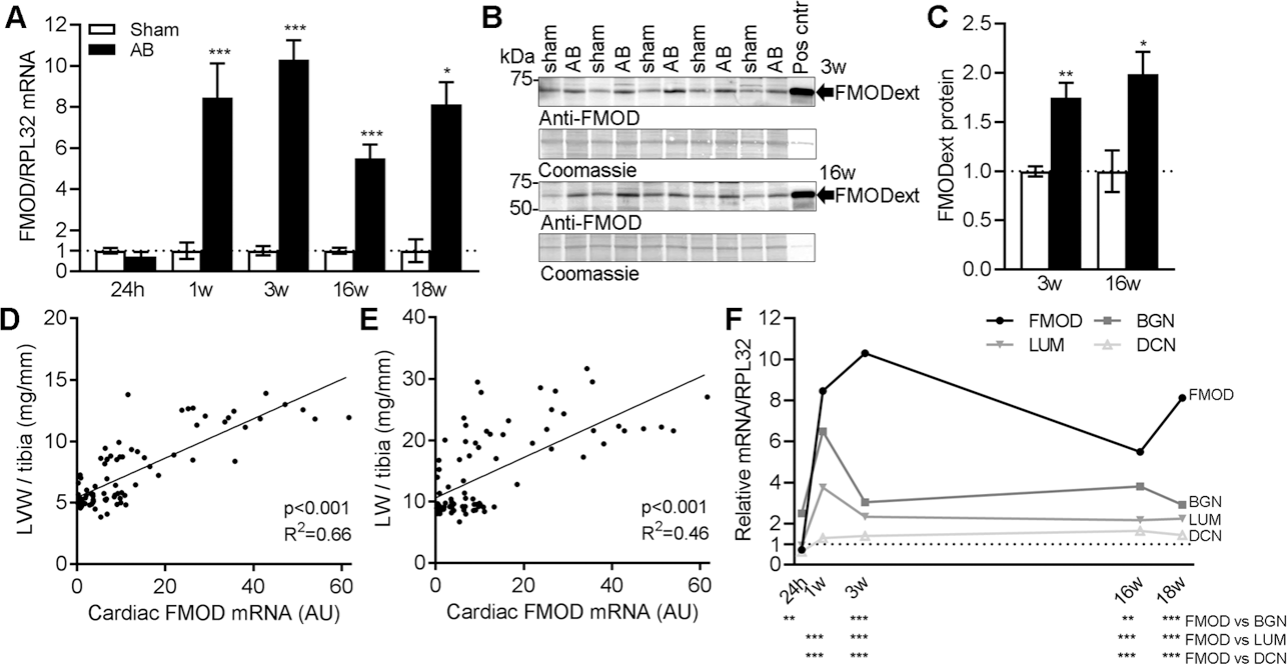
Fibromodulin is upregulated in hearts of mice in response to pressure overload. (A) FMOD mRNA in the left ventricle (LV) of wild-type (WT) mice subjected to aortic banding (AB) for 24 hours (h) -18 weeks (w), compared to sham-operated controls, n sham = 3–10, n AB = 7–10. Ribosomal protein L32 (RPL32) was used as reference gene. (B) Representative immunoblots and (C) quantitative levels of extracellular FMOD (FMODext, ~60kDa) protein in mouse LVs 3w and 16w post-AB or sham operation, n = 5–7. Culture medium of FMOD-expressing HEK293 cells was used as positive control (Pos cntr), see S1 Fig for details. Coomassie staining was used for loading control. (D and E) Pearson regression analysis of FMOD mRNA and (D) left ventricular weight (LVW)/tibia length, or (E) lung weight (LW)/tibia length in sham- and AB-operated mice, n = 87. (F) FMOD, lumican (LUM), biglycan (BGN) and decorin (DCN) mRNA normalized to RPL32 in LVs of mice 24h-18w post-AB, relative to the average of sham-operated controls, n sham = 3–10, n AB = 7–10. Mouse characteristics are found in S4 Table. Data are shown as mean±SEM. Statistical differences were tested using an unpaired t-test vs. respective sham-operated controls (A and C), Pearson regression analysis (D and E) and one-way ANOVA with Bonferroni post-hoc test vs. FMOD mRNA (F). *p≤0.05; ** p≤0.01; ***p≤0.005.

To investigate the FMOD expression dynamics in HF progression, we measured cardiac mRNA and protein levels of FMOD in the WT cohort subjected to AB for 18w. We examined the cardiac phenotype after AB by echocardiography, and harvested LVs during the acute phase (i.e. 24 hours (h) post-AB), during hypertrophic remodeling (1-3w post-AB) and during end-stage, dilated HF (16-18w post-AB) (mouse characteristics are found in S4 Table). FMOD mRNA was unaltered at 24h post-AB (Fig 2A). FMOD mRNA was increased 8-, 10-, 6- and 8-fold at 1, 3, 16 and 18w post-AB compared to controls, also confirmed by increased FMOD protein levels (Fig 2B and 2C). FMOD mRNA correlated positively with LV weight and lung weight, suggesting that higher FMOD levels were associated with more severe LV remodeling and congestive HF (Fig 2D and 2E, respectively). Of notice, FMOD expression increased more than the expression of other SLRPs with known functions in the heart, i.e. LUM, BGN and DCN (Fig 2F).

### Fibromodulin is expressed in cardiomyocytes and cardiac fibroblasts and regulated by inflammatory pathways

**Fig 3:**
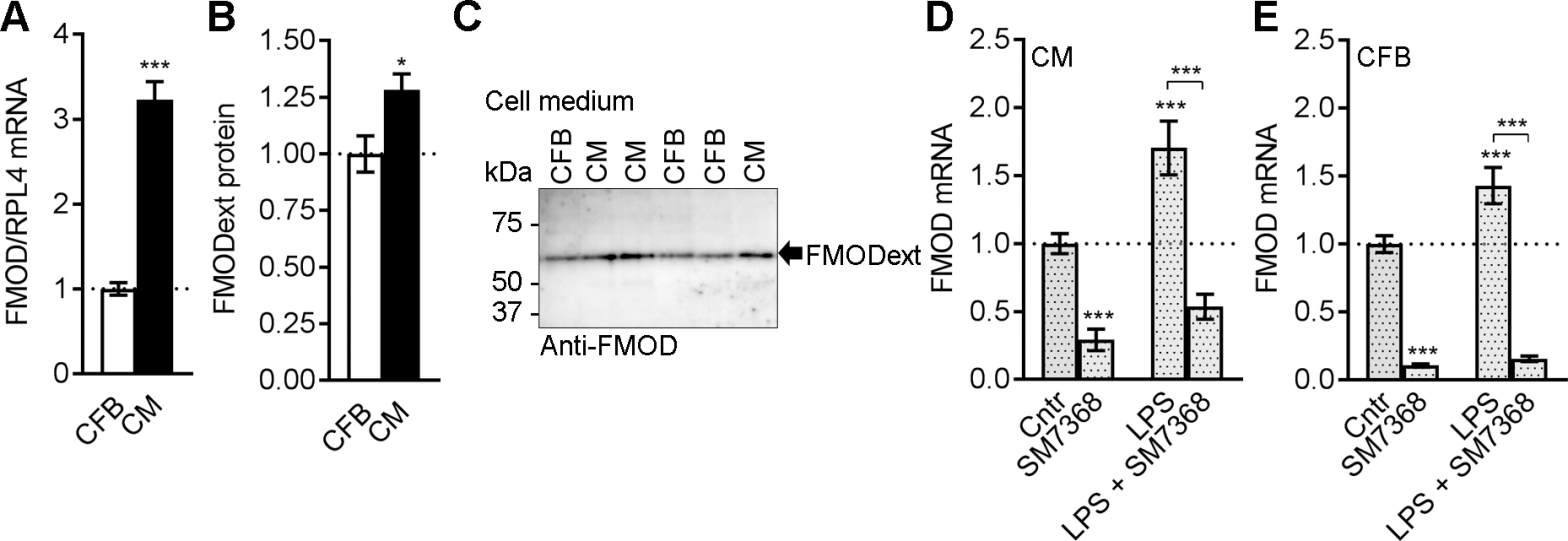
Fibromodulin is expressed in cardiomyocytes and cardiac fibroblasts and regulated by inflammatory pathways. (A) FMOD mRNA in cultured cardiomyocytes (CM) and cardiac fibroblasts (CFB) from neonatal rats, n = 10–11. Ribosomal protein L4 (RPL4) was used as reference gene. (B) Quantitative levels of extracellular FMOD (FMODext) protein in culture medium from CM and CFB, n = 6–10, with (C) representative immunoblots. (D) CM and (E) CFB cultures were treated with the innate immunity mediator lipopolysaccharide (LPS) or the nuclear factor (NF)κB-inhibitor SM7368, alone or in combination. CM, n = 9–18, and CFB, n = 10–19. Data are shown as mean±SEM. Statistical differences were tested using an unpaired t-test, CM vs. CFB (A-B), *p≤0.05; ***p≤0.005, and (D-E) one-way ANOVA with Holm-Sidak post-hoc test vs. Cntr or co-treatment with SM7368 vs. LPS alone, *** p≤0.005.

We assessed FMOD levels in the two major cell types in the heart, CFB and CM. Cultured CM showed a 3-fold mRNA expression of FMOD compared to CFB (Fig 3A). Consistently, we found 1.3-fold levels of FMODext protein secreted to the medium from CM compared to CFB (Fig 3B and 3C). Thus, FMOD was expressed in both CFB and CM.

To investigate if pro-inflammatory pathways regulate FMOD expression in cardiac cells, CFB and CM were treated with a mediator of innate immunity, LPS, alone or in combination with the NFκB inhibitor SM7368. SM7368 attenuated the basal FMOD expression in both cell types, while LPS treatment upregulated FMOD (Fig 3D and 3E). Co-treatment with SM7368 attenuated the LPS-induced FMOD increase. These data are consistent with a role for FMOD in inflammation, suggesting its expression is regulated by the NFκB transcription factor.

### FMOD-KO mice show no cardiac phenotype without induced cardiac stress

FMOD-KO mice are viable, fertile and have an appearance similar to that of respective WT controls [15]. Genotyping confirmed WT, heterozygous and FMOD-KO offspring from heterozygous intercrosses (A in S2 Fig). As expected, we found no FMOD mRNA (B in S2 Fig) or protein (C-D in S2 Fig) in hearts of FMOD-KO mice, and we did not detect dysregulation of other SLRPs with known function in the heart, i.e. LUM, BGN and DCN (B and E-F in S2 Fig). Adult, untreated mice (7-9w of age, baseline) showed no echocardiographic phenotype, with blood pressure, body-, heart-, and lung weight similar to that of controls (S5 Table).

### FMOD-KO mice show mildly exacerbated hypertrophic remodeling after cardiac pressure overload

**Fig 4:**
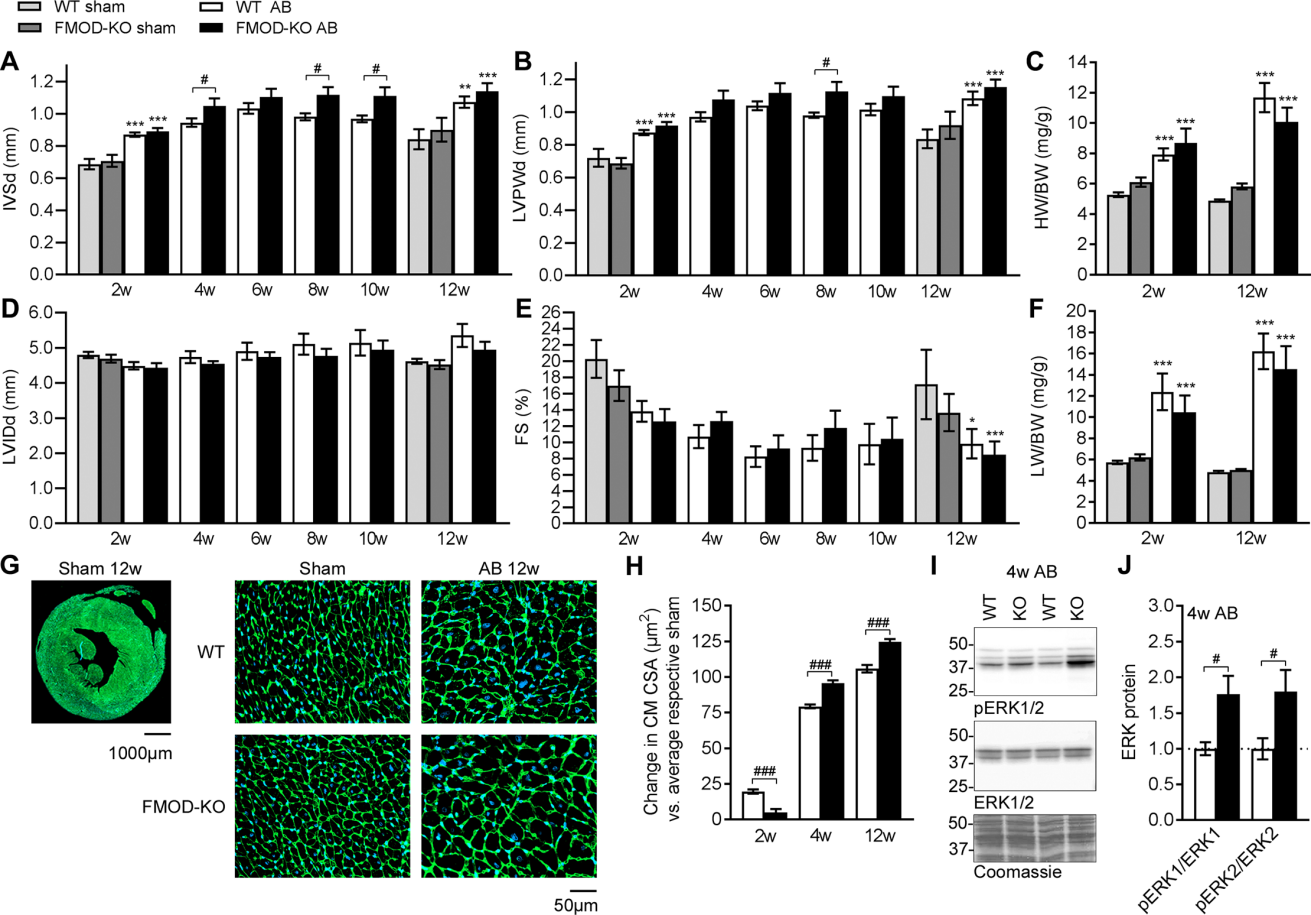
FMOD-KO mice show mildly exacerbated hypertrophic remodeling compared to wild-type, associated with increased ERK signaling upon aortic banding. Cardiac phenotype assessed by serial echocardiography at 2–12 weeks (w) (A-B and D-E, n sham = 5–10, n AB = 5–24) and organ weights at 2w and 12w (C and F, n sham = 5–14, n AB = 9–14) post aortic banding (AB). (A) Interventricular septum thickness in diastole (IVSd), (B) left ventricular posterior wall thickness in diastole (LVPWd), (C) heart weight-to-body weight (HW/BW) ratio, (D) LV interior diameter in diastole (LVIDd), (E) fractional shortening (FS) and (F) lung weight-to-body weight (LW/BW) ratio in fibromodulin knock-out (FMOD-KO) and wild-type (WT) control mice subjected to AB. (G) Representative histology image of a whole heart section at low magnification(scale bar 1000μm) and insets showing representative histology images at high magnification (scale bar 50μm) of the different groups at 12w post operation, green (WGA staining) shows outline of cardiomyocyte cross sectional area (CSA) and blue (DAPI staining) shows nucleus. (H) Quantitative changes in cardiomyocyte (CM) cross-sectional area (CSA) respective to average sham controls, measured on midventricular sections of FMOD-KO and WT mice 2, 4, and 12w post-AB, n = 2191–14485 CMs from n sham = 1–4 mice, and n AB = 2–4 mice. (I) Representative immunoblots and (J) quantitative phosphorylated (p) and total levels of the extracellular signal–regulated kinase 1 and 2 (ERK1 and ERK2, respectively) in LVs of FMOD-KO and WT mice 4w post-AB (n sham = 2, n AB = 3–5). Coomassie staining was used as loading control. Data are shown as mean±SEM. Statistical differences were tested using an unpaired t-test vs. WT AB, #p≤0.05; ###p≤0.005 (A, B, D and E 4-10w, H and J), one-way ANOVA with Dunn's post-hoc test vs. WT sham, *p≤0.05; **p≤0.01; ***p≤0.005, or vs. WT AB (A-F 2w and 12w).

To assess whether FMOD plays a role in cardiac remodeling following pressure overload we compared cardiac dimensions and function in FMOD-KO to WT control mice subjected to pressure overload. There was no difference in mortality between the two genotypes post-AB (S3 Fig). Both genotypes showed hypertrophic growth with thicker septum and LV posterior wall compared to sham controls after AB (Fig 4A and 4B). FMOD-KO mice showed thicker septum than WT controls at 4, 8 and 10w post-AB, and thicker LV posterior wall at 8w, i.e. FMOD-KO mice showed mildly exacerbated hypertrophic remodeling in response to pressure overload. Heart weight was increased in both genotypes compared to sham at 2w and 12w post-AB, however we found no difference between FMOD-KO and WT control mice post-AB (Fig 4C). During progression of remodeling from 2-12w post-AB, hearts were not dilated compared to controls (Fig 4D). Contractile function, assessed as fractional shortening (FS), was similarly decreased in both genotypes at 12w post-AB and compatible with severe HF (Fig 4E). EF was 26% and 23% in WT AB and FMOD-KO AB (p = 0.02 and p = 0.004, vs. 43% in WT sham), respectively, at 12w. These EF values show HF with a comparable severity to the patient cohort (average EF of 19%). As for FS, there were no differences in EF between WT AB and FMOD-KO AB. Lung weight was increased in both genotypes compared to sham at 2w and 12w post-AB, but to a similar extent in FMOD-KO and WT (Fig 4F), also suggesting comparable degree of congestive HF after AB. Expression of HF signature molecules atrial and brain natriuretic peptides (encoded by NPPA and NPPB, respectively) were increased after AB, as expected (A-C in S4 Fig), however levels were comparable in FMOD-KO and WT mice 2, 4, or 12w post-AB, consistent with similar degree of cardiac stress and HF. To further pursue the role of FMOD in cardiac hypertrophy, we assessed CM size histologically on midventricular sections of FMOD-KO and WT hearts post-AB. Consistent with increased wall thickness on echocardiography, CM from FMOD-KO hearts showed a larger increase in CSA compared to WT, both at 4w and 12w after AB (Fig 4G and 4H). Expression of major sarcomere genes, including myosins (encoded by MYH6 and MYH7) was not significantly different between genotypes at 2, 4, or 12w post-AB (A-C in S4 Fig). Assessing pro-hypertrophic signaling, we found increased cardiac ERK1/2 phosphorylation (i.e. activation) in FMOD-KO mice post-AB (Fig 4I and 4J). Thus, the mildly exacerbated hypertrophic remodeling observed in FMOD-KO mice was associated with increased ERK signaling.

To test whether there was a direct effect of FMOD on CM hypertrophy, FMOD was overexpressed in cultured CMs. Increased FMOD mRNA was confirmed in CM transduced with adenovirus encoding FMOD (AdFMOD), with no effects on expression of the other SLRPs LUM, BGN and DCN (A in S5 Fig). However, FMOD overexpression did not affect CM growth *in vitro*, assessed as protein synthesis by radioactive leucine incorporation (A in S6 Fig), and had no major effect on expression of sarcomere genes (B in S6 Fig). Thus, the effect of FMOD on CM hypertrophy was likely indirect.

### FMOD-KO mice show no alteration in cardiac fibrosis compared to wild-type after pressure overload

**Fig 5:**
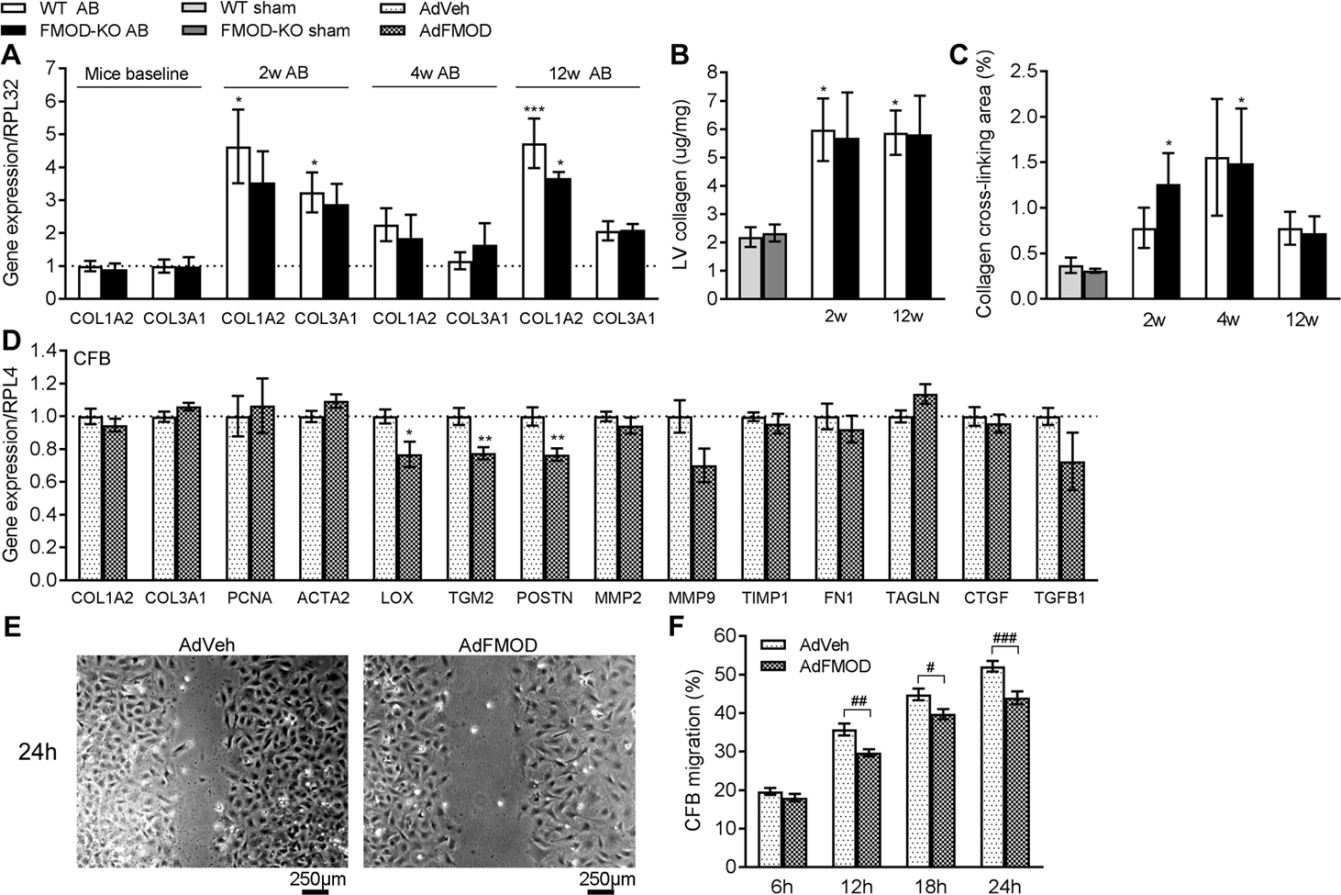
FMOD-KO and wild-type mice have comparable levels of fibrillar collagens and collagen cross-linking in vivo, but fibromodulin decreases the migration and alters the expression of fibrosis-associated genes in cultured cardiac fibroblasts in vitro. (A) COL1A2 and COL3A1 mRNA in the left ventricle (LV) of fibromodulin knock-out (FMOD-KO) and wild-type (WT) mice at baseline, 2, 4, or 12 weeks (w) post-aortic banding (AB), relative to average of sham-operated controls (n sham = 2–10, n AB = 3–8). Ribosomal protein L32 (RPL32) was used as reference gene. (B) Collagen protein levels assessed by HPLC from whole LV, in mice 2w and 12w post-AB or sham-operation, n sham = 5, n AB = 5–6. (C) Amount of collagen cross-linking quantified from short axis midventricular histological sections of hearts of FMOD-KO and WT mice 2, 4 and 12w after sham or AB operation, n sham = 5–7, n AB = 2–4. (D) mRNA expression of collagens (COL1A2, COL3A1), cell signature proliferation marker PCNA, myofibroblast differentiation signature marker ACTA2, and fibrosis-associated transcripts in cardiac fibroblast (CFB) cultures from neonatal rats transduced with adenovirus encoding FMOD (AdFMOD), or adenovirus-vehicle (AdVeh) as control, n = 6–7. Ribosomal protein L4 (RPL4) was used as reference gene. (E) Representative images of CFB with AdVeh or AdFMOD 24h post-scratching, and (F) quantification of CFB migration 6-24h post-scratching, n = 8–10. Scale bar 250μm. Data are shown as mean±SEM. Statistical differences were tested using one-way ANOVA with Dunn's post-hoc test vs. WT sham, *p≤0.05; ***p≤0.005, or vs. WTAB (A, B and C), unpaired t-test vs. AdVeh, *p≤0.05; **p≤0.01 (D), or two-way ANOVA (F), #p≤0.05; ##p≤0.01; ###p≤0.005.

To assess whether FMOD affects cardiac fibrosis, fibrillar collagen levels were measured in FMOD-KO hearts post-AB. We did not detect any differences in COL1A2 (encoding collagen I) and COL3A1 (encoding collagen III) mRNA levels (Fig 5A) in FMOD-KO and WT mice at baseline. As expected, expression of COL1A2 mRNA (Fig 5A) and collagen protein levels, measured by high-performance liquid chromatography (HPLC) from whole LV (Fig 5B), were increased in WT mice 2 and 12w post-AB compared to sham. However, we found no differences in collagen levels comparing FMOD-KO to WT 2 and 12w post-AB. To address whether FMOD affected cardiac collagen cross-linking, midventricular histological cross-sections of FMOD-KO and WT hearts were assessed. Collagen cross-linking was increased in FMOD-KO mice hearts 2 and 4w post-AB compared to respective sham controls (Fig 5C). However, in WT mice the increase post-AB did not reach statistical significance. Thus, FMOD-KO mice showed no differences in cardiac fibrosis compared to WT post-AB.

### Fibromodulin regulates migration and expression of fibrosis-associated proteins in cultured cardiac fibroblasts

To assess whether FMOD affected the pro-fibrotic properties of CFB, fibrillar collagen levels were measured in CFB overexpressing FMOD *in vitro*. Increased FMOD mRNA, without dysregulation of the other SLRPs LUM, BGN and DCN, was confirmed in CFB transduced with AdFMOD (B in S5 Fig). In accordance with the finding of no difference in fibrillar collagen expression in FMOD-KO hearts, CFB expression of COL1A2 and COL3A1 was unaffected by FMOD overexpression (Fig 5D).

To address whether FMOD affected CFB properties central to fibrosis, expression of the signature molecule of cardiac fibroblast-to-myofibroblast transdifferentiation (ACTA2, encoding α-skeletal muscle actin) and a molecular marker of proliferation (PCNA, encoding proliferating cell nuclear antigen) were measured, and a migration scratch assay was performed. Expression of ACTA2 or PCNA was unaltered in CFB overexpressing FMOD (Fig 5D). However, FMOD decreased the migration of CFB after scratching (Fig 5E and 5F). These results indicated that FMOD directly affects migration of CFB, without effects on proliferation or myofibroblast transdifferentiation.

To test whether FMOD could have a direct effect on collagen cross-linking, mRNA expression of the collagen cross-linking enzyme lysyl oxidase (encoded by LOX) and transglutaminase 2 (encoded by TGM2) were measured in CFB transduced with AdFMOD. Interestingly, overexpression of FMOD reduced the expression of LOX and TGM2 in CFB (Fig 5D). Furthermore, reduced expression of the ECM protein periostin (encoded by POSTN, Fig 5D) suggested a direct effect of FMOD on the pro-fibrotic properties of CFB. We found no differences in levels of TGFβ (Fig 5D). Thus, FMOD reduced the expression of the fibrosis-associated molecules lysyl oxidase, transglutaminase 2, and periostin.

Finally, we did not observe differences in expression of LOX, TGM2 or POSTN in hearts of FMOD-KO and WT mice at 2, 4, or 12w post-AB (S7 Fig).

### FMOD-KO mice show attenuated cardiac immune cell infiltration after pressure overload

**Fig 6:**
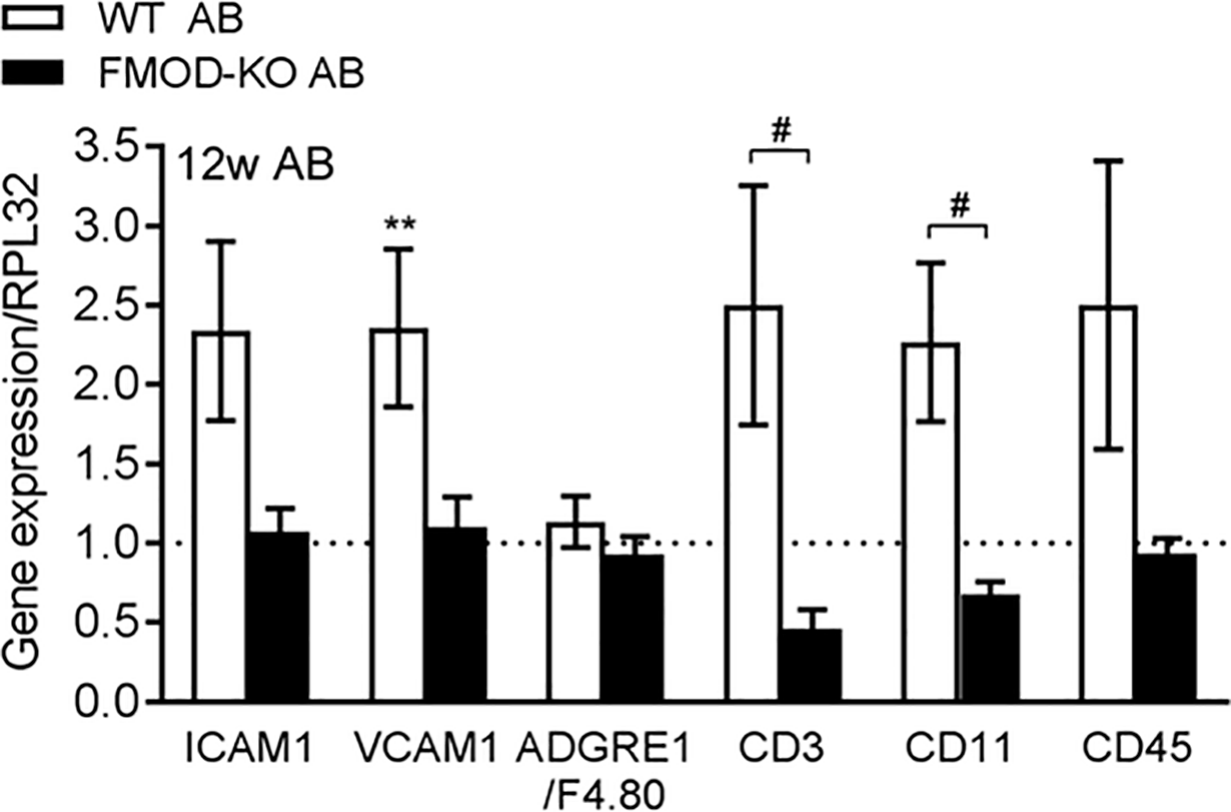
Fibromodulin affects the cardiac immune response after pressure overload. mRNA expression of immune cell adhesion molecules (ICAM1 and VCAM1) and signature molecules of macrophages (ADGRE1/F4.80), T-lymphocytes (CD3), or leukocytes (CD11a and CD45) in the left ventricle of FMOD knock-out (FMOD-KO) and wild-type (WT) mice 12 weeks (w) post-aortic banding (AB), relative to respective sham-operated controls, n sham = 10, n AB = 4–7. Ribosomal protein L32 (RPL32) was used as reference gene. Data are shown as mean±SEM. Statistical differences were tested using one-way ANOVA with Dunn's post-hoc test vs. WT sham, **p≤0.01, or vs. WT AB, #p≤0.05.

To address whether FMOD affected inflammation, immune cell infiltration was assessed *in vivo* by measuring expression of central immune cell adhesion molecules (ICAM1 and VCAM1, encoding intercellular adhesion molecule-1 and vascular cell adhesion molecule 1, respectively), and signature molecules of leukocytes (CD45, encoding cluster of differentiation 45 and CD11a, encoding cluster of differentiation 11), T-cells (CD3, encoding cluster of differentiation 3), and macrophages (ADGRE1/F4.80, encoding adhesion G protein-coupled receptor E1) in FMOD-KO and WT LV post-AB. We found no immune cell infiltration at 2w or 4w post-AB compared to sham controls (S8 Fig). Interestingly, infiltration of leukocytes, including T-cells, was evident in WT mice 12w post-AB but not in FMOD-KO mice (Fig 6). Thus, our results suggest that FMOD regulates inflammation and immune cell infiltration in the pressure-overloaded heart.

## Discussion

**Fig 7:**
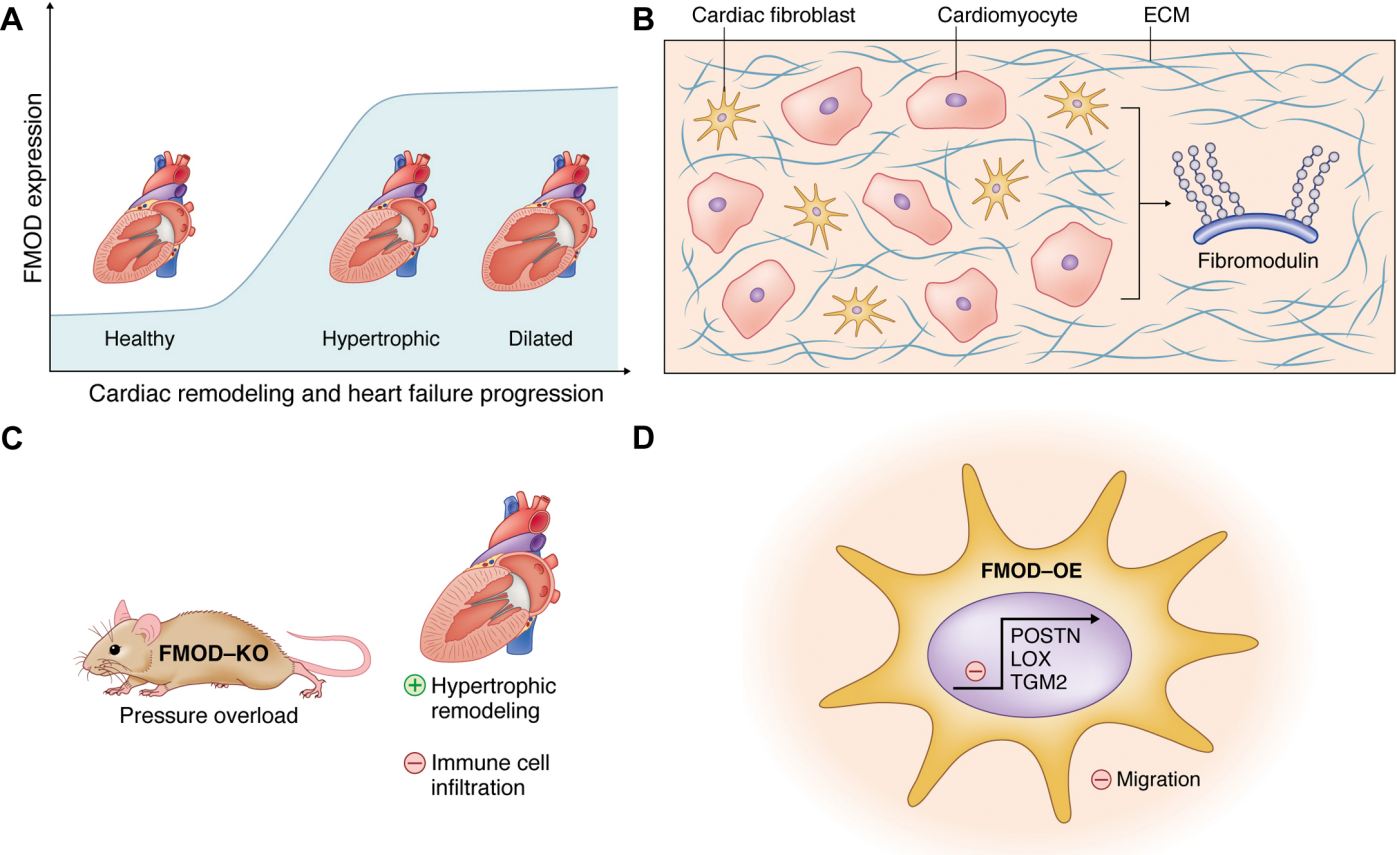
Schematic illustration of the proposed role of fibromodulin in the failing heart. The schematic illustrates the main findings of our study. (A) Fibromodulin (FMOD) expression in the heart is increased during cardiac remodeling and heart failure progression in patients and mice. (B) FMOD is an extracellular matrix (ECM) proteoglycan produced by both cardiomyocytes and cardiac fibroblasts, the two major cell types in the heart. (C) FMOD knock-out (FMOD-KO) mice show mildly exacerbated hypertrophic remodeling of the heart with attenuated infiltration of leukocytes in response to experimental pressure overload. (D) FMOD overexpression (FMOD-OE) in cultured cardiac fibroblasts decreases their migratory capacity and reduces the expression of fibrosis-related ECM molecules such as periostin (POSTN), transglutaminase 2 (TGM2), and the collagen-cross linking enzyme lysyl oxidase (LOX). Thus, we propose that FMOD has anti-hypertrophic, anti-fibrotic and pro-inflammatory effects in the pressure-overloaded heart.

We investigated expression levels of FMOD in HF, studied the role of FMOD in hearts by subjecting FMOD-KO mice to pressure overload, and examined properties of cultured cardiac cells after FMOD overexpression. We found that FMOD was upregulated in the myocardium of patients and mice with HF. Both CM and CFB produced FMOD, and its expression was increased upon pro-inflammatory stimuli. Pressure overload of FMOD-KO mice induced mildly exacerbated hypertrophic remodeling with increased pro-hypertrophic ERK1/2 signaling and attenuated infiltration of leukocytes. When overexpressed in CFB, FMOD reduced the migratory capacity and the expression of fibrosis-related ECM molecules. The proposed role of FMOD in the failing heart is illustrated in Fig 7.

Our finding of increased FMOD levels in experimental and clinical HF is in line with recent bioinformatics data on differentially expressed genes in myocardial tissue from patients with hypertrophic and dilated cardiomyopathy [25]. The up-regulation of FMOD was stronger (6-10-fold in AB mice) than that of other SLRPs with a known role in the heart, i.e. LUM, BGN, DCN, suggesting that FMOD has a particularly important role among SLRPs in the failing heart. Thus, we investigated the cardiac phenotype of FMOD-KO mice.

CFBs are believed to be the principal cells responsible for production of ECM proteins [31, 32]. Unexpectedly, we found that both CM and CFB express FMOD and that CM produce more FMOD than CFB. It is therefore likely that both cell types contribute to the increased levels of FMOD in HF, and that FMOD could directly affect the properties of CM as well as CFB. This formed the basis for our FMOD overexpression experiments in cultures of both cell types.

A central, and surprising finding based on the robust up-regulation in HF, was the relatively mild cardiac phenotype of FMOD-KO mice upon pressure overload. Without stress, FMOD-KO mice have similar cardiac phenotype to controls, and upon AB, FMOD-KO mice show mildly exacerbated hypertrophic remodeling with increased wall thickness and CM size, without effects on dilatation, contractile function, pulmonary congestion, expression of major HF or remodeling markers, or mortality. In comparison, the proteoglycan syndecan-4 is up-regulated 3-fold after AB in mice, yet syndecan-4 KO mice show a strong phenotype with attenuated hypertrophy, exacerbated dilatation, contractile dysfunction and pulmonary congestion post-AB [10, 11, 33, 34]. LUM and FMOD compete for binding to collagen fibrils [35–37], and LUM-KO mice show a stronger cardiac phenotype than FMOD-KO mice with 50% perinatal death and hypertrophic growth from the neonatal stage in surviving mice [38]. Double-LUM/FMOD-KO mice show clinical features of Ehler-Danlos syndrome with joint laxity and impaired tendon integrity [39]. In tendon, it has been suggested that increased LUM compensates for the lack of FMOD in FMOD-KO mice [15, 39], however since LUM levels were unaffected in FMOD-KO hearts, we do not believe that LUM compensated for lack of FMOD in the heart.

The hypertrophic phenotype of the FMOD-KO mice was associated with increased ERK1/2 signaling. ERK1/2 are central signaling kinases in pathological hypertrophy [40], and increased activation in FMOD-KO hearts is in line with our finding of increased hypertrophy.

Immune cell infiltration to the heart affects remodeling [41]. A central finding in this study is that immune cell infiltration to the FMOD-KO heart was attenuated upon AB. These results are in line with the finding that FMOD activates and sustains the inflammatory response in cartilage [42]. We speculate that the mild hypertrophic phenotype of FMOD-KO mice post-AB could result from dysregulated inflammation or attenuated immune cell infiltration. Our finding from cultured CM showing that FMOD overexpression had no direct effect on CM hypertrophy could support that FMOD affects hypertrophy through altered tissue infiltration of inflammatory cells, that are absent in CM cultures. Also, we found that FMOD expression was increased in cultured CM and CFB through the LPS-NFκB axis in CM and CFB, supporting a role for FMOD in cardiac inflammation and innate immunity pathways. Evidence supports a role for the innate immunity in HF progression [43, 44], and indeed, expression of proteoglycans such as syndecans, lumican and versican, are elevated through activation of innate immunity [22, 28, 45].

Accumulation of fibrillar collagens in the ECM is a hallmark of heart fibrosis [7]. FMOD is widely distributed in connective tissues and affects collagen fibrillogenesis [9]. FMOD-KO mice exhibit fewer and disorganized collagen fibrils with abnormal morphology in tail tendon [15], teeth [9, 46], the sclera of the eye [47], and skin [48]. Another characteristic feature of SLRPs, including FMOD, is their ability to bind collagens [9, 10, 13]. SLRP coating of collagen fibrils protects them from proteolysis by limiting the access of collagenases [9, 13]. Thus, we hypothesized that fibrosis was altered in FMOD-KO hearts, however we found no differences in fibrillar collagen amount. This is in contrast to proteoglycans such as syndecan-4 and lumican that regulates cardiac collagen expression and deposition in the heart [22, 27, 34, 49].

Alongside increased deposition of fibrillar collagens, collagen cross-linking stiffens the matrix and contributes to cardiac dysfunction [31, 50]. SLRP coating of collagen fibrils regulates the lateral association of collagens into fibrils [9, 13]. In mouse tendons, FMOD binds to collagen cross-linking sites and activates lysyl oxidase, the major enzyme responsible for collagen cross-linking [51]. We therefore hypothesized that collagen cross-linking was affected in FMOD-KO hearts, but found no differences. Nevertheless, overexpression of FMOD in CFB resulted in reduced expression of LOX. The amount of lysyl oxidase in cardiac tissue correlates with degree of collagen cross-linking [50], and thus, we speculate that the increased cardiac FMOD levels in HF could contribute to reducing the stiffness of the matrix through down-regulation of LOX.

Proliferation, migration and activation of CFB is central to cardiac fibrosis [32]. We show that increased FMOD levels reduces the migration of CFB, which is in line with previous findings showing that FMOD affects skin fibroblast migration during wound healing [48].

Numerous molecular players are involved in development of cardiac fibrosis [4]. We found that increased CFB level of FMOD reduces the expression of transglutaminase 2 and periostin. Periostin is a well-known downstream target of TGFβ, and a major player in fibrosis and remodeling after pressure overload [52, 53]. Our finding that increased FMOD levels reduces the levels of periostin in CFB are in line with findings from skin scars, where FMOD regulates fibrotic responses through interaction with TGFβ [48]. Overexpression of TGM2 results in cardiac fibrosis [54]. Thus, that increased FMOD reduces CFB levels of the fibrosis-related molecules periostin and transglutaminase 2 suggests that FMOD has anti-fibrotic effects.

Targeting cardiac fibrosis is attractive therapeutically [6, 8]. TGFβ and TGFβ-directed molecules such as periostin, lysyl oxidase and transglutaminase 2 [50, 55] represent potential therapeutic targets in limiting or reversing cardiac fibrosis. As such, further investigations into the role of FMOD in attenuating CFB migration and reducing the expression of pro-fibrotic molecules are warranted. Recent studies show that FMOD is essential for the fetal-type scarless wound healing in skin [21], and that intradermal administration of FMOD reduces scar size, increases the tensile strength and improves the dermal collagen organization in skin wounds of rodents and pigs [20, 56]. Thus, FMOD-based therapies show translational potential in cutaneous wound repair and could possibly also hold promise in treatment of cardiac fibrosis.

In conclusion, our study adds to the growing body of evidence supporting an important role for proteoglycans in cardiac remodeling and HF [5, 10]. We show that FMOD is robustly upregulated in HF and affects hypertrophic remodeling and immune cell infiltration in response to pressure overload. Finally, FMOD affects CFBs properties that impact on cardiac fibrosis.

## Supporting information

S1 Fig Overexpression of fibromodulin in HEK293 cells. (DOCX)

S2 Fig No expression of fibromodulin in fibromodulin knock-out mice. (DOCX)

S3 Fig No difference in survival between fibromodulin knock-out and wild-type mice after aortic banding. (DOCX)

S4 Fig No major differences in expression of natriuretic peptides or cardiomyocyte sarcomere genes in hearts of fibromodulin knock-out and wild-type mice after aortic banding. (DOCX)

S5 Fig Overexpression of fibromodulin in cultured cardiomyocytes and cardiac fibroblasts. (DOCX)

S6 Fig No major differences in natriuretic peptides or sarcomere genes in cardiomyocytes overexpressing fibromodulin. (DOCX)

S7 Fig No major differences in expression of fibrosis-associated genes in hearts of wild-type and fibromodulin knock-out mice after aortic banding. (DOCX)

S8 Fig No immune cell infiltration in hearts of mice 2–4 weeks post-aortic banding. (DOCX)

S1 Table Assays used to determine gene expression. (DOCX)

S2 Table Primary antibodies with specifications used for immunoblotting. (DOCX)

S3 Table Characteristics of end-stage heart failure patients. (DOCX)

S4 Table Characteristics of aortic-banded wild-type mouse cohort. (DOCX)

S5 Table Baseline characteristics of fibromodulin knock-out (FMOD-KO) and wild-type (WT) controls in adult, untreated mice. (DOCX)

S6 Table Raw data. (XLSX)

## Abbreviations

AB: aortic banding
ACTA2: α-skeletal muscle actin
AdFMOD: adenovirus encoding fibromodulin
ADGRE1/F4.80: adhesion G protein-coupled receptor E1
BGN: biglycan
CD11: cluster of differentiation 11
CD3: cluster of differentiation 3
CD45: cluster of differentiation 45
CFB: cardiac fibroblast
CM: cardiomyocyte
CSA: cross-sectional area
DCN: decorin
ECM: extracellular matrix
EF: ejection fraction
ERK: extracellular signal-regulated kinase
FMOD: fibromodulin
FMOD-KO: fibromodulin knock-out
FS: fractional shortening
GAG: glycosaminoglycan
HF: heart failure
ICAM: intercellular adhesion molecule
IVSd: interventricular septal thickness in diastole
LOX: lysyl oxidase
LPS: lipopolysaccharide
LRR: leucine-rich repeat
LUM: lumican
LV: left ventricle
LVIDd: left ventricular internal diameter in diastole
LVIDs: left ventricular internal diameter in systole
LVPWd: left ventricular posterior wall thickness in diastole
PCNA: proliferating cell nuclear antigen
POSTN: periostin
SLRP: small leucine-rich proteoglycan
TGF: transforming growth factor
TGM2: transglutaminase 2
VCAM: vascular cell adhesion molecule
WT: wild-type
