# Supplementary material for: The extracellular matrix proteoglycan fibromodulin is upregulated in clinical and experimental heart failure and affects cardiac remodeling
Source: PLoS One. 2018 Jul 27;13(7):e0201422. doi: 10.1371/journal.pone.0201422 (PMC6063439; doi:10.1371/journal.pone.0201422)
Supplement: S1 Fig — (DOCX) [file pone.0201422.s001.docx]

**
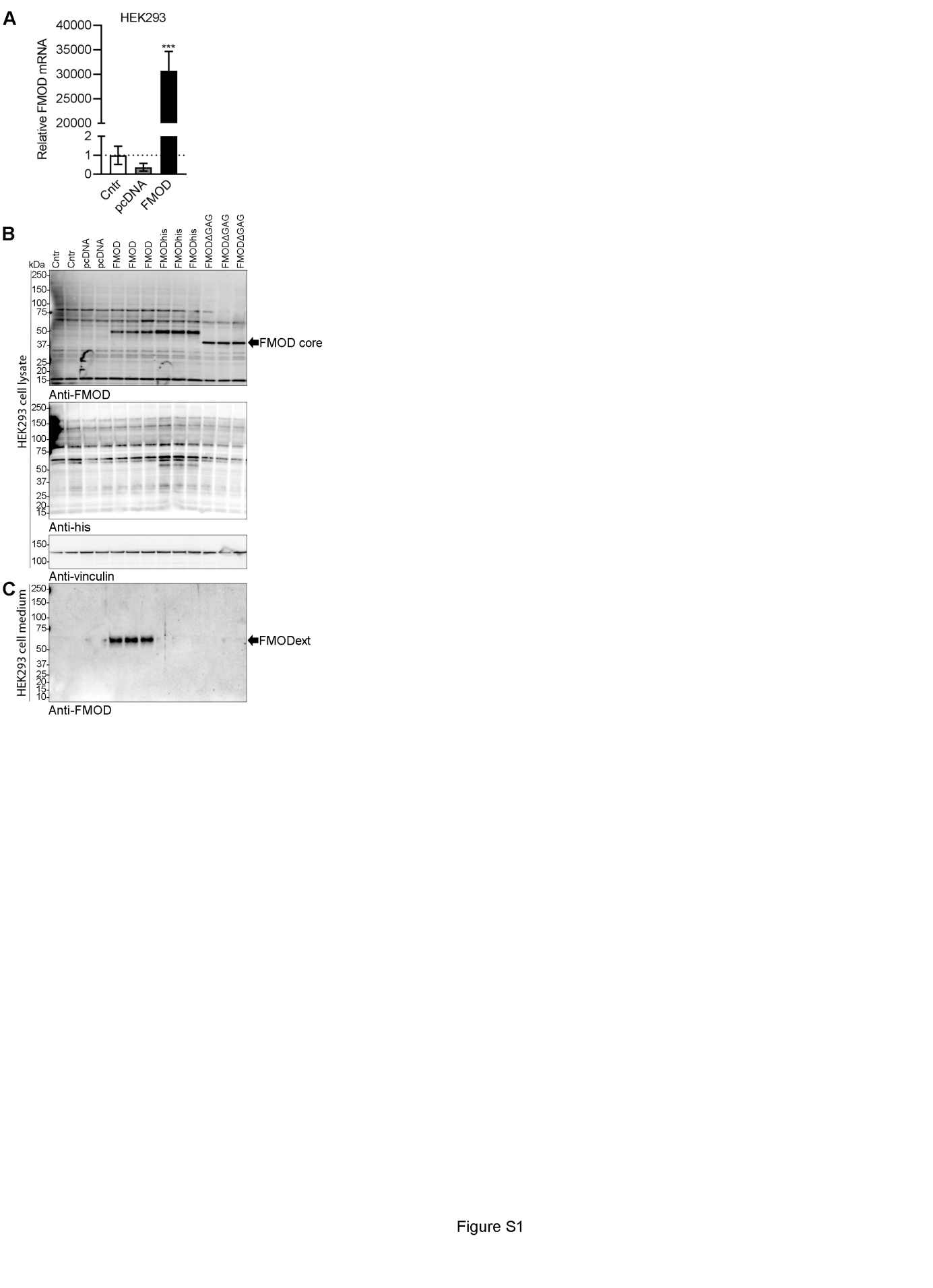
**

**S1 Fig. Overexpression of fibromodulin in HEK293 cells.**

Human endothelial kidney (HEK) 293 cells were transfected with plasmids encoding human FMOD, His-tagged FMOD (FMODHis) or a FMOD mutant (FMODΔGAG) where glycosaminoglycan (GAG) attachment sites in FMOD were mutated (N127A, N166A, N201A, N291A, N341A). (A) FMOD mRNA (n=3-6), (B and C) representative immunoblots of HEK293 cell lysate (B) and culture medium (C), n=3, in FMOD-transfected HEK293 cells, vehicle-transfected (pcDNA) and non-transfected (Cntr) controls. The non-glycosylated FMOD core protein (FMOD core) was detected at the expected 42 kDa in FMODΔGAG samples. The intracellular FMOD with and without his-tag (FMODhis and FMOD, respectively) were detected at ~50 kDa. FMOD was secreted into the cell medium at ~60kDa, representing the extracellular FMOD (FMODext). Cell medium containing FMODext was used as positive control for immunoblotting of FMOD throughout our study. The his-tagged FMOD and FMODΔGAG were not secreted into the cell medium. Anti-his immunoblotting was performed after stripping of the anti-FMOD blot. Anti-vinculin was used for loading control of cell lysates. Data are shown as mean±SEM. Statistical differences were tested using one-way ANOVA with Dunnett's post-hoc test, ***p≤0.005.
