# Supplementary material for: The extracellular matrix proteoglycan fibromodulin is upregulated in clinical and experimental heart failure and affects cardiac remodeling
Source: PLoS One. 2018 Jul 27;13(7):e0201422. doi: 10.1371/journal.pone.0201422 (PMC6063439; doi:10.1371/journal.pone.0201422)
Supplement: S2 Fig — (DOCX) [file pone.0201422.s002.docx]

**
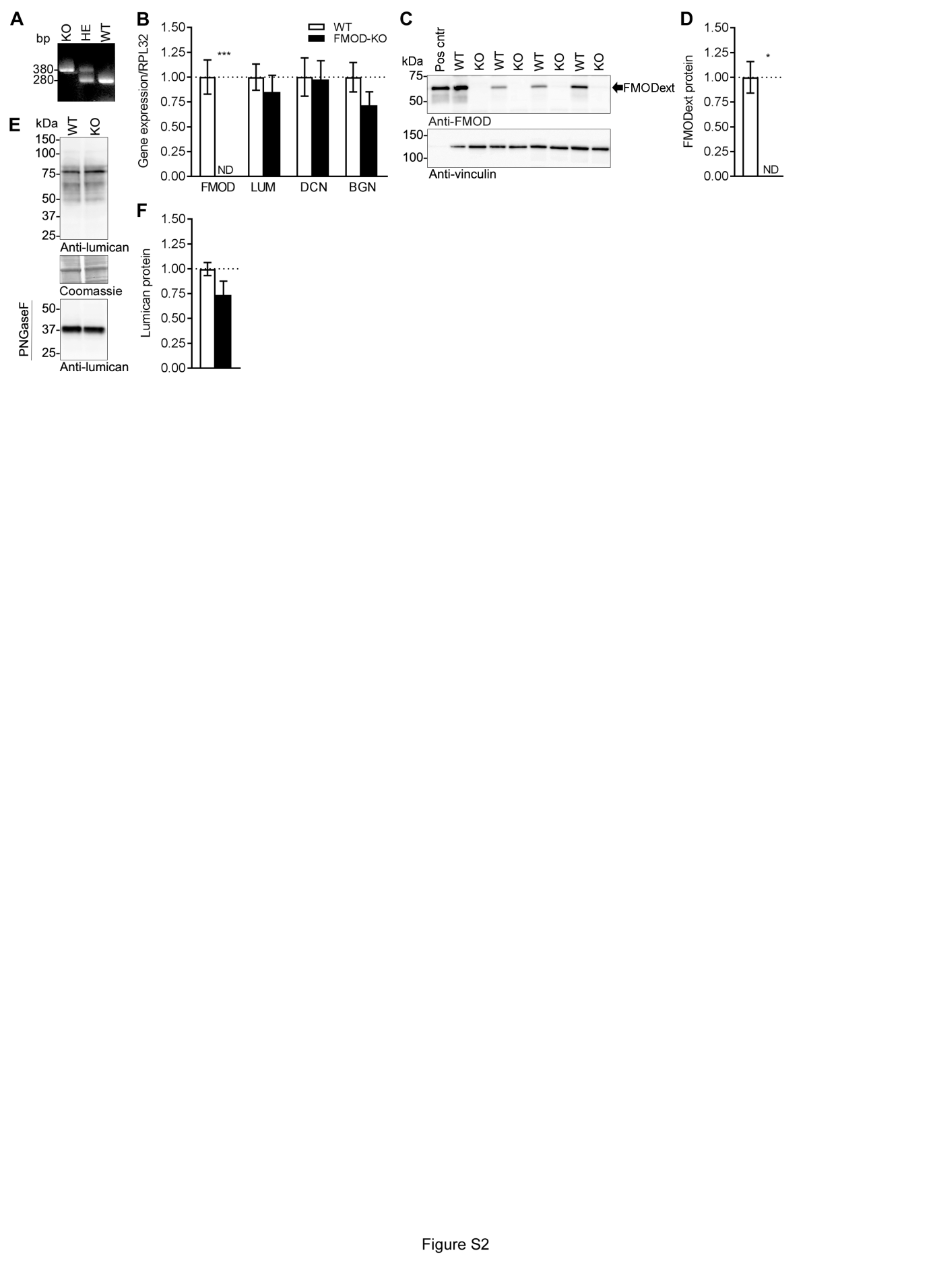
**

**S2 Fig. No expression of fibromodulin in fibromodulin knock-out mice.**

(A) Representative genotyping gel of DNA from ear biopsies from homo (KO)- and heterozygous (HE) fibromodulin knock-out (FMOD-KO) mice and wild-type (WT) littermates at weaning age, showing the expected WT band at 280 bp and KO band at 380 bp [1]. Fibromodulin, lumican, decorin and biglycan are small leucine rich proteoglycans (SLRPs). (B) mRNA of FMOD, LUM, DCN, and BGN in the left ventricle (LV) of WT and FMOD-KO mice at baseline, n=5-8. Ribosomal protein L32 (RPL32) was used as reference gene. (C) Representative immunoblots and (D) quantification of extracellular FMOD (FMODext, ~60kDa) protein in the LV of FMOD-KO and WT mice at baseline, n=4. Cell medium from FMOD-transfected HEK293 cells was used as positive control (Pos cntr), see S1 Fig. Vinculin was used as loading control. (E) Representative immunoblots and (F) quantification of PNGaseF-treated samples of lumican protein in FMOD-KO and WT mice at baseline, n=4. Upper blot shows untreated LV samples with lumican detected at 50-75 kDa. Coomassie staining was used for loading control. Lower blot shows samples treated with PNGaseF with deglycosylated lumican detected at the expected 38 kDa [2]. Data are shown as mean±SEM. Statistical differences were tested using t-test vs. WT, *p≤0.05; ***p≤0.005. ND, not detected.

**References**

1. Svensson L, Aszodi A, Reinholt FP, Fassler R, Heinegard D, Oldberg A. Fibromodulin-null mice have abnormal collagen fibrils, tissue organization, and altered lumican deposition in tendon. J Biol Chem. 1999;274(14):9636-47.

2. Engebretsen KV, Lunde IG, Strand ME, Wæhre A, Sjaastad I, Marstein HS, et al. Lumican is increased in experimental and clinical heart failure, and its production by cardiac fibroblasts is induced by mechanical and proinflammatory stimuli. The FEBS journal. 2013;280(10):2382-98.
