# Supplementary material for: The extracellular matrix proteoglycan fibromodulin is upregulated in clinical and experimental heart failure and affects cardiac remodeling
Source: PLoS One. 2018 Jul 27;13(7):e0201422. doi: 10.1371/journal.pone.0201422 (PMC6063439; doi:10.1371/journal.pone.0201422)
Supplement: S3 Fig — (DOCX) [file pone.0201422.s003.docx]

**
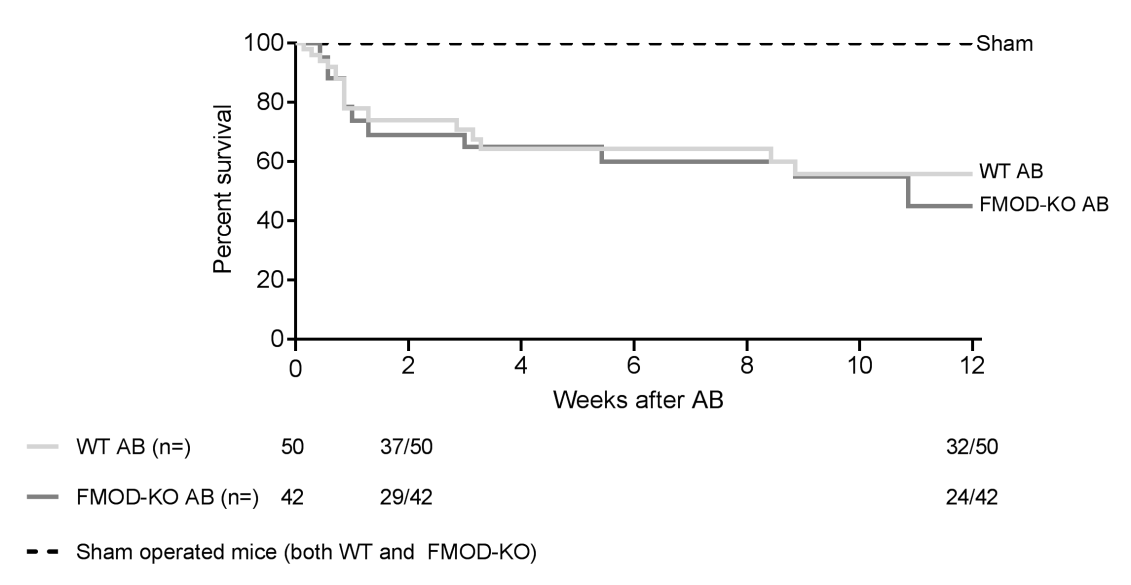
**

**S3 Fig. No difference in survival between fibromodulin knock-out and wild-type mice after aortic banding.**

Kaplan-Meier survival plot shows % survival of fibromodulin knock-out (FMOD-KO) and wild-type (WT) control mice up to 12 weeks after aortic banding (AB) or sham operation. All sham-operated FMOD-KO and WT mice survived throughout the study. Numbers of surviving animals at baseline, 2- and 12 weeks after AB are noted under graph, n sham=19-26. Statistical differences were tested using logrank test vs. WTAB.
