# Supplementary material for: The extracellular matrix proteoglycan fibromodulin is upregulated in clinical and experimental heart failure and affects cardiac remodeling
Source: PLoS One. 2018 Jul 27;13(7):e0201422. doi: 10.1371/journal.pone.0201422 (PMC6063439; doi:10.1371/journal.pone.0201422)
Supplement: S4 Fig — (DOCX) [file pone.0201422.s004.docx]

**
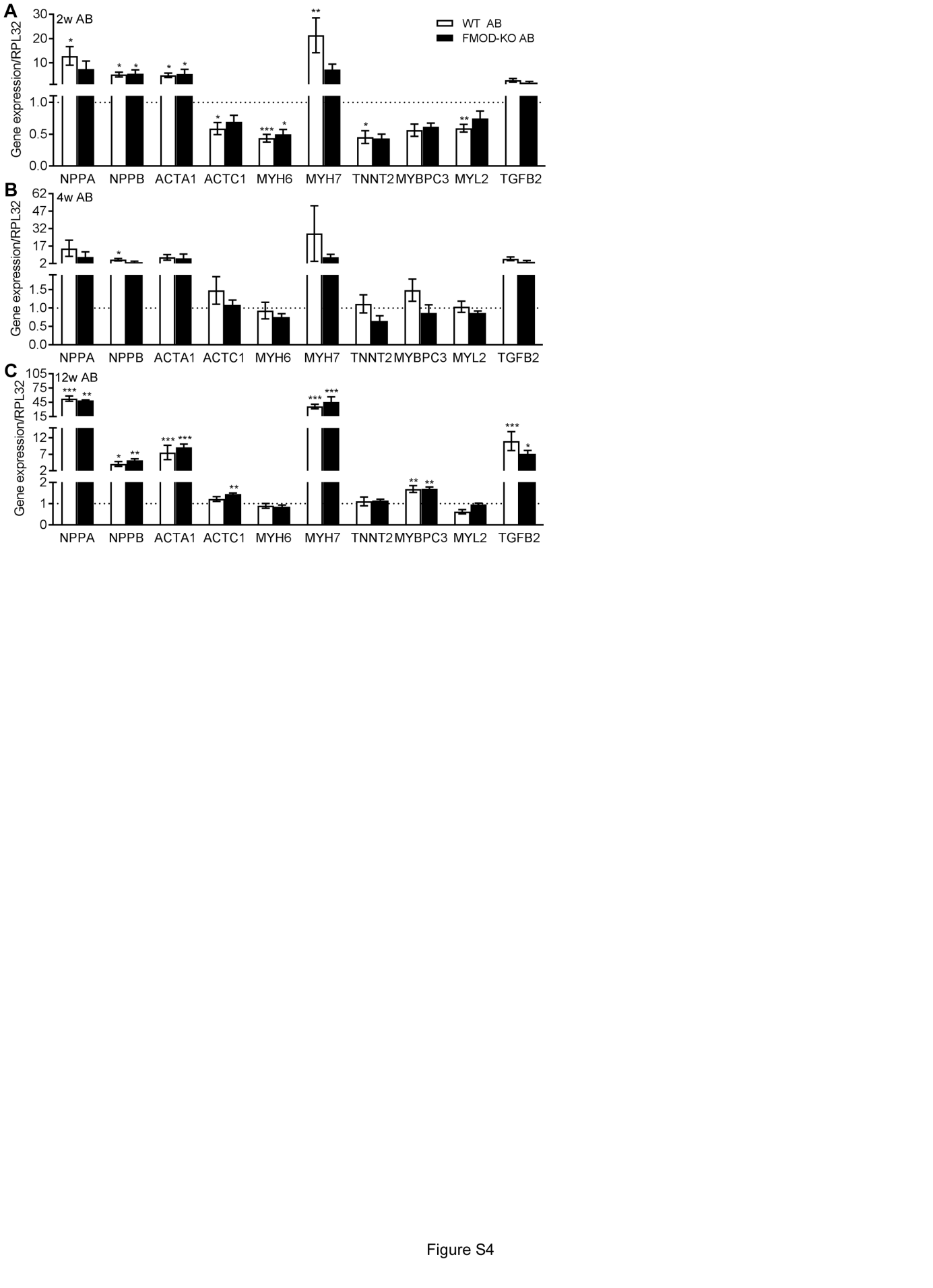
**

**S4 Fig. No major differences in expression of natriuretic peptides or cardiomyocyte sarcomere genes in hearts of fibromodulin knock-out and wild-type mice after aortic banding**. mRNA expression of cardiomyocyte-specific and hypertrophy-associated transcripts in the left ventricle (LV) of fibromodulin knock-out (FMOD-KO) and wild-type (WT) mice at 2w, 4w, and 12w post-aortic banding (AB) (A-C, respectively), relative to WT sham-operated controls set to 1, n sham = 2-10, n AB = 3-8. Ribosomal protein L32 (RPL32) was used as reference gene. Data are shown as mean±SEM. Statistical differences were tested by one-way ANOVA with Dunn's post-hoc test vs. WT sham, *p≤0.05; **p≤0.01; ***p≤0.005, or vs. WT AB (no detected differences).
