# Supplementary material for: The extracellular matrix proteoglycan fibromodulin is upregulated in clinical and experimental heart failure and affects cardiac remodeling
Source: PLoS One. 2018 Jul 27;13(7):e0201422. doi: 10.1371/journal.pone.0201422 (PMC6063439; doi:10.1371/journal.pone.0201422)
Supplement: S5 Fig — (DOCX) [file pone.0201422.s005.docx]

**
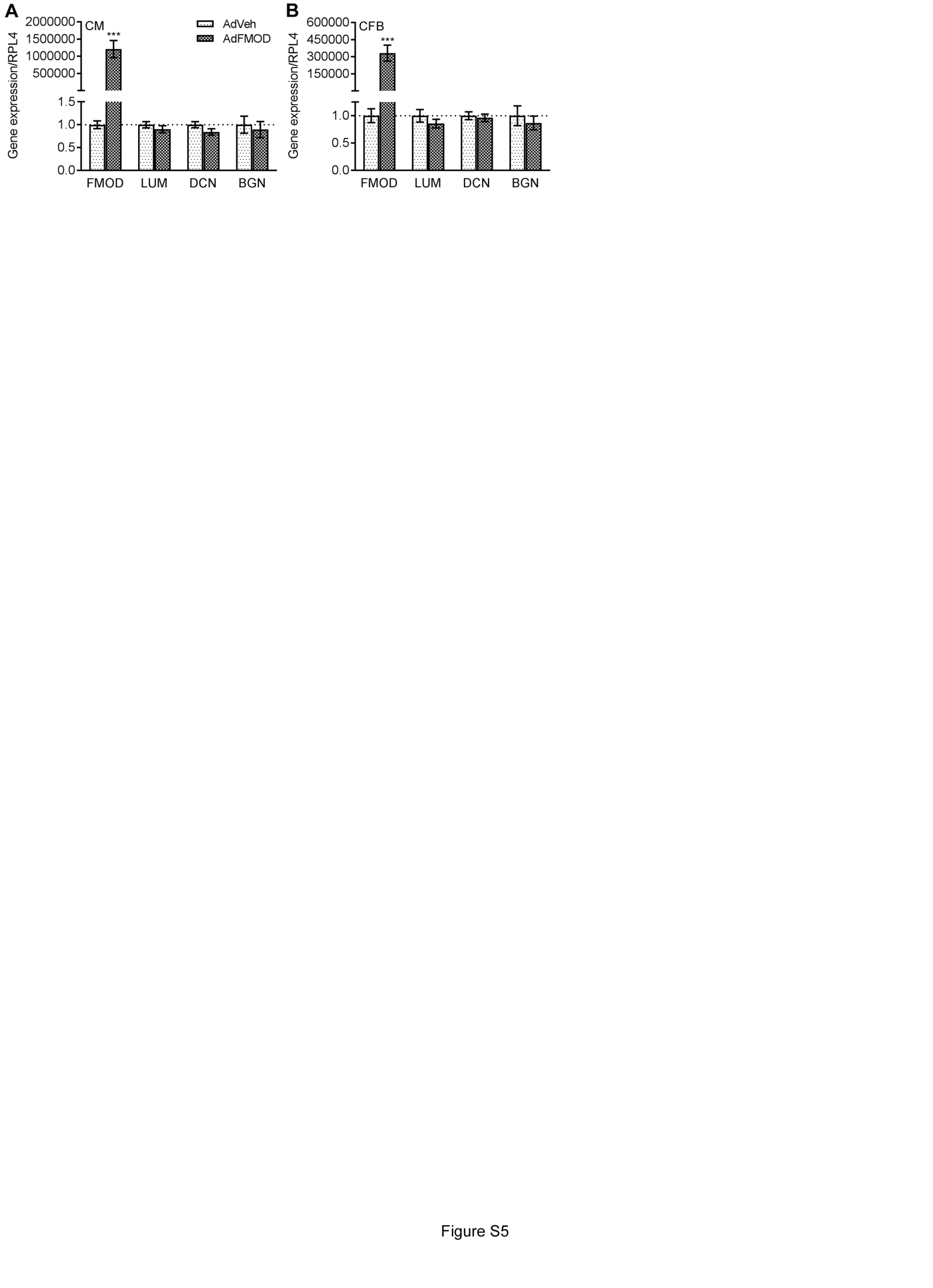
**

**S5 Fig. Overexpression of fibromodulin in cultured cardiomyocytes and cardiac fibroblasts.** (A) Cardiomyocyte (CM) and (B) cardiac fibroblast (CFB) cultures from neonatal rats were transduced with an adenovirus encoding FMOD (AdFMOD), or adenovirus-vehicle (AdVeh) as control. Fibromodulin, lumican, decorin and biglycan are

small leucine rich proteoglycans (SLRPs). mRNA expression of FMOD, LUM, DCN and BGN in CM and CFB overexpressing FMOD, n=7-9. Ribosomal protein L4 (RPL4) was used as reference gene. Data are shown as mean±SEM. Statistical differences were tested using an unpaired t-test vs. AdVeh set to 1, ***p≤0.005.
