# Supplementary material for: The extracellular matrix proteoglycan fibromodulin is upregulated in clinical and experimental heart failure and affects cardiac remodeling
Source: PLoS One. 2018 Jul 27;13(7):e0201422. doi: 10.1371/journal.pone.0201422 (PMC6063439; doi:10.1371/journal.pone.0201422)
Supplement: S6 Fig — (DOCX) [file pone.0201422.s006.docx]

**
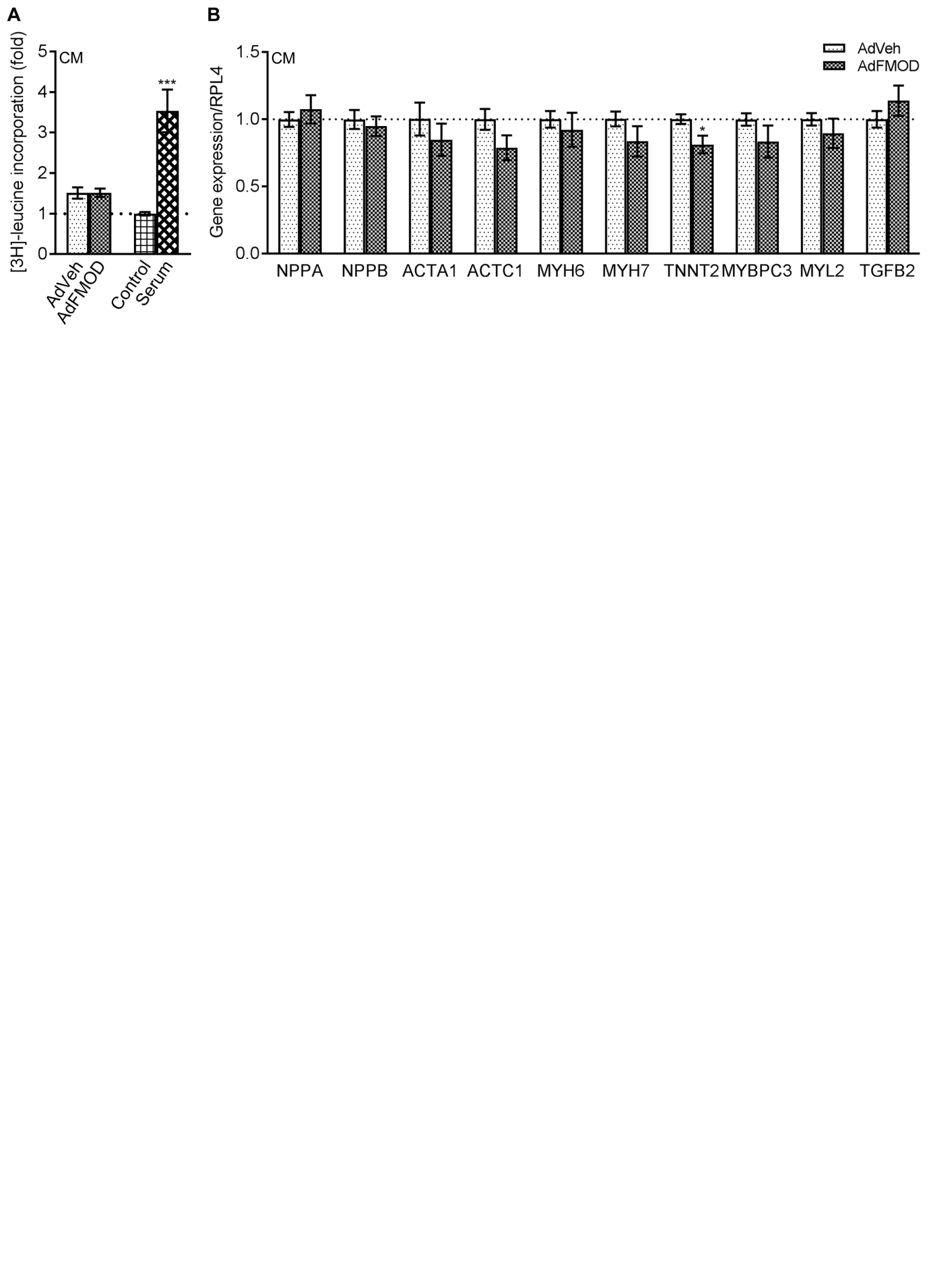
**

**S6 Fig. No major differences in natriuretic peptides or sarcomere genes in cardiomyocytes overexpressing fibromodulin. (**A) Cardiomyocyte (CM) cultures from neonatal rats were transduced with an adenovirus encoding FMOD (AdFMOD), or adenovirus-vehicle (AdVeh) as control, n=18-34. CM growth was assessed as protein synthesis measured by radioactive leucine incorporation. Serum was used as positive control relative to serum-starved controls set to 1, n=9. (B) mRNA expression of natriuretic peptides and sarcomere genes was measured in cardiomyocyte (CM) cultures from neonatal rats transduced with an adenovirus encoding FMOD (AdFMOD), or adenovirus-vehicle (AdVeh) as control set to 1, n=9. Ribosomal protein L4 (RPL4) was used as reference gene. Data are shown as mean±SEM. Statistical differences were tested using an unpaired t-test vs. AdVeh, *p≤0.05, or vs. control, ***p≤0.005.
