# Supplementary material for: The extracellular matrix proteoglycan fibromodulin is upregulated in clinical and experimental heart failure and affects cardiac remodeling
Source: PLoS One. 2018 Jul 27;13(7):e0201422. doi: 10.1371/journal.pone.0201422 (PMC6063439; doi:10.1371/journal.pone.0201422)
Supplement: S8 Fig — (DOCX) [file pone.0201422.s008.docx]

**
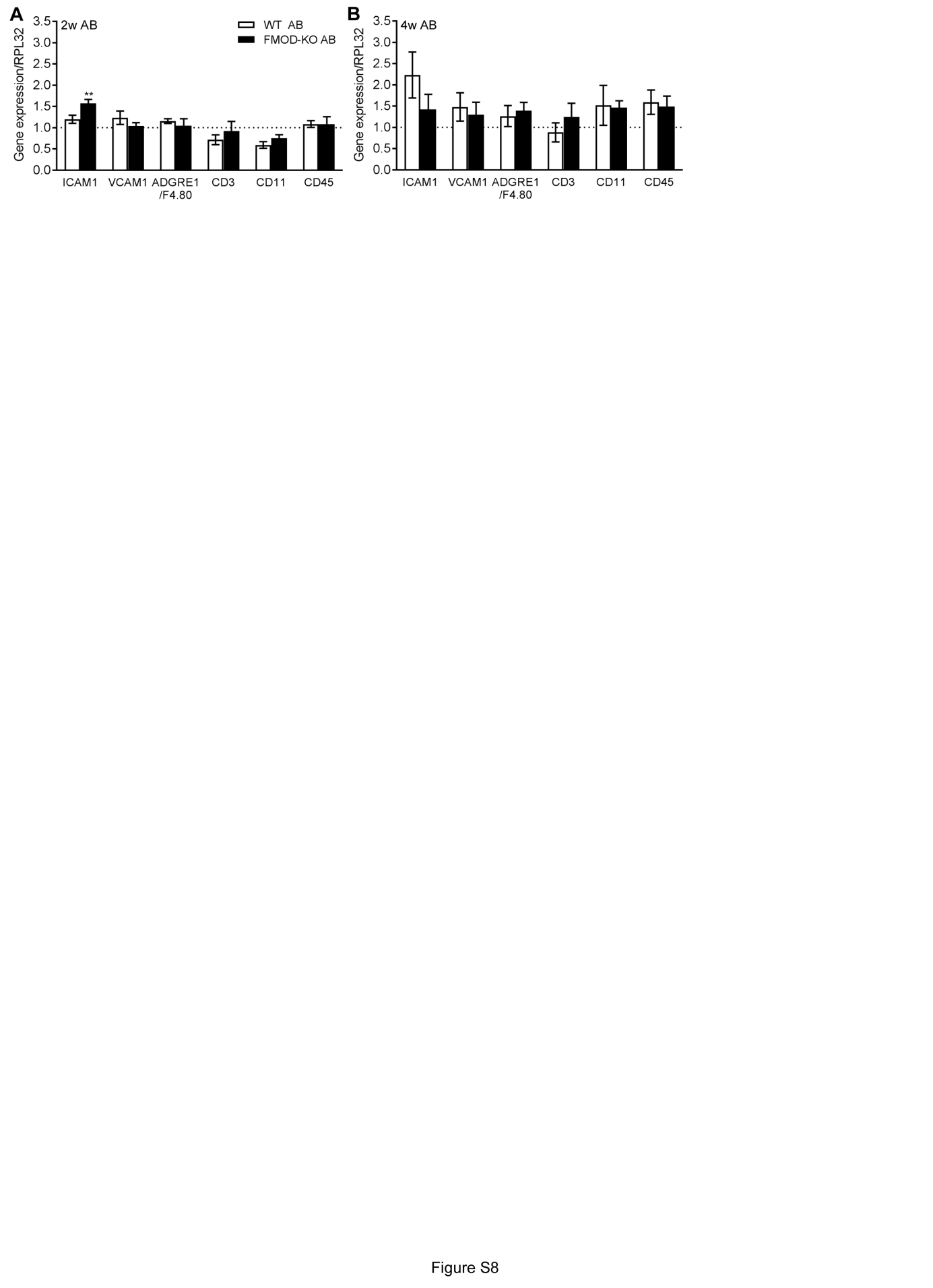
**

**S8 Fig. No immune cell infiltration in hearts of mice 2-4 weeks after aortic banding.** mRNA expression of immune cell adhesion molecules (ICAM1 and VCAM1) and signature molecules of macrophages (ADGRE1/F4.80), T-lymphocytes (CD3), or leukocytes (CD11a and CD45) in the left ventricle of fibromodulin knock-out (FMOD-KO) and wild-type (WT) mice at (A) 2 and (B) 4 weeks (w) post-aortic banding (AB), relative to average of WT sham-operated controls set to 1, n sham=2-7, n AB=3-8. Ribosomal protein L32 (RPL32) was used as reference gene. Data are shown as mean±SEM. Statistical differences were tested using one-way ANOVA with Dunn's post-hoc test vs. WT sham, *p≤0.05; **p≤0.01, or vs. WT AB (no detected differences).
