# Supplementary material for: The extracellular matrix proteoglycan fibromodulin is upregulated in clinical and experimental heart failure and affects cardiac remodeling
Source: PLoS One. 2018 Jul 27;13(7):e0201422. doi: 10.1371/journal.pone.0201422 (PMC6063439; doi:10.1371/journal.pone.0201422)
Supplement: S1 Table — (DOCX) [file pone.0201422.s009.docx]

**S1 Table. Assays used to determine gene expression.**

| **Gene/transcript** | **Mouse assays** | **Rat assays** | **Human assays** | **Protein:** |
| --- | --- | --- | --- | --- |
| COL1A2 | Mm00483888_m1 | Rn01526721_m1 |  | collagen type I alpha chain |
| COL3A1 | Mm01254476_m1 | Rn01437681_m1 |  | collagen type III alpha chain |
| FN1 | Mm01256744_m1 | Rn00569575_m1 |  | fibronectin |
| ACTA2 | Mm01546133_m1 | Rn01759928_g1 |  | alpha smooth muscle actin |
| TAGLN | Mm00441660_m1 | Rn01642285_g1 |  | transgelin |
| LOX | Mm00495386_m1 |  |  | lysyl oxidase |
| POSTN | Mm01284919_m1 | Rn01494627_m1 |  | periostin |
| TGM2 | Mm00436987_m1 | Rn00571440_m1 |  | transglutaminase 2 |
| MMP2 | Mm00439498_m1 | Rn01538177_m1 |  | matrix metalloproteinase-2 |
| MMP9 | Mm00579162_m1 | Rn00579162_m1 |  | matrix metalloproteinase-9 |
| TIMP1 | Mm00441818_m1 | Rn01430873_g1 |  | metallopeptidase inhibitor 1 |
| CTGF | Mm01192933_g1 | Rn01537279_g1 |  | connective tissue growth factor |
| TGFβ1 | Mm01178820_m1 | Rn00572010_m1 |  | transforming growth factor beta 1 |
| TGFβ2 | Mm004336955_m1 | Rn00579674_m1 |  | transforming growth factor beta 2 |
| NPPA | Mm01255747_g1 | Rn00664637_g1 |  | atrial natriuretic peptide |
| NPPB | Mm01255770_g1 | Rn00580641_m1 |  | brain natriuretic peptide |
| ACTA1 | Mm00808218_g1 | Rn01426628_g1 |  | skeletal muscle alpha actin |
| ACTC1 | Mm01333821_m1 | Rn01513700_g1 |  | cardiac muscle alpha actin |
| MYH6 | Mm00440359_m1 | Rn00691721_g1 |  | myosin heavy chain alpha |
| MYH7 | Mm00600555_m1 | Rn00568328_m1 |  | myosin heavy chain beta |
| TNNT2 | Mm01290256_m1 |  |  | cardiac muscle troponin T2 |
| MYBPC3 | Mm00435104_m1 | Rn01513426_m1 |  | cardiac myosin binding protein C3 |
| MYL2 | Mm00440384_m1 | Rn01480558_g1 |  | myosin light chain 2 |
| FMOD | Mm00491215_m1 | Rn00589918_m1 | Hs00157619_m1 | fibromodulin |
| LUM | Mm01248292_m1 | Rn00579127_m1 |  | lumican |
| DCN | Mm00514535_m1 | Rn01503161_m1 |  | decorin |
| BGN | Mm01191753_m1 | Rn00567229_m1 |  | biglycan |
| ICAM1 | Mm00516023_m1 | Rn00564227_m1 |  | intercellular adhesion molecule 1 |
| VCAM1 | Mm01320970_m1 | Rn00563627_m1 |  | vascular cell adhesion protein 1 |
| ADGRE1/F4.80 | Mm00802529_m1 |  |  | adhesion G protein-coupled receptor E1 |
| CD3 | Mm01179194_m1 |  |  | cluster of differentiation 3 |
| CD11a | Mm00801807_m1 |  |  | cluster of differentiation 11 |
| CD45 | Mm01293577_m1 |  |  | cluster of differentiation 45 |
| RPL32 | Mm02528467_g1 | Rn00820748_g1 | Hs00851655_g1 | ribosomal protein L4 |
| RPL4 | Mm00834993_g1 | Rn00821091_g1 | Hs03044646_g1 | ribosomal protein L30 |

Pre-designed TaqMan assays used to determine gene expression by qPCR. Gene expression was normalized to ribosomal protein L4 (RPL4) or L32 (RPL32).
