# Supplementary material for: The extracellular matrix proteoglycan fibromodulin is upregulated in clinical and experimental heart failure and affects cardiac remodeling
Source: PLoS One. 2018 Jul 27;13(7):e0201422. doi: 10.1371/journal.pone.0201422 (PMC6063439; doi:10.1371/journal.pone.0201422)
Supplement: S2 Table — (DOCX) [file pone.0201422.s010.docx]

**S2 Table. Primary antibodies with specifications used for immunoblotting.**

| **Protein** | **Antibody** |
| --- | --- |
| Fibromodulin | NBP1_31657 (1:750), Novus Biotech |
| Lumican | AF2745 (1:500), R&D Systems |
| His | A00186-100 (1:5000), Genscript |
| pERK1/2 | #9101 (1:1000), Cell Signaling Technology |
| ERK1/2 | #9102 (1:1000), Cell Signaling Technology |
| Vinculin | #V9131 (1:1000000), Sigma |

Primary antibodies used for immunoblotting. ERK1/2, extracellular signal–regulated kinase 1 and 2 (ERK1/2), phospho (p). Vinculin or Coomassie staining were used for loading control.
