# Supplementary material for: The extracellular matrix proteoglycan fibromodulin is upregulated in clinical and experimental heart failure and affects cardiac remodeling
Source: PLoS One. 2018 Jul 27;13(7):e0201422. doi: 10.1371/journal.pone.0201422 (PMC6063439; doi:10.1371/journal.pone.0201422)
Supplement: S3 Table — (DOCX) [file pone.0201422.s011.docx]

**S3 Table. Characteristics of end-stage heart failure patients.**

|  | **HF (n=17)** |
| --- | --- |
| Age (years) | 49.4 ± 2.8 |
| Gender | 3 females, 14 males |
| LVEF (%) | 19.2 ± 1.6 (all <35%) |
| NYHA class | 8 NYHA III, 9 NYHA IV |
| BMI (kg/m^2^) | 26.6 ± 0.7 |
| Pro-BNP (pmol/L) | 541.3 ± 145.9 |
| LVEDV (ml) | 287.7 ± 22.7 |
| LVESV (ml) | 235.5 ± 21.5 |
| SV (ml) | 51.7 ± 3.8 |
| IVSd (cm) | 0.8 ± 0.1 |
| LVPWd (cm) | 0.8 ± 0.0 |
| LVIDd (cm) | 7.5 ± 0.2 |

Clinical and echocardiographic data (mean±SEM) of patients with end-stage heart failure (HF) with reduced ejection fraction (HFrEF, n=17) from whom left ventricular (LV) biopsies were taken from their explanted hearts. Left ventricular biopsies from non-diseased donor hearts considered for transplantation, but deemed unsuitable, were used as controls (n=5, age 39.4±7.8 years, 3 females). LVEF, LV ejection fraction; NYHA, New York Heart Association; BMI, body mass index; BNP, brain natriuretic peptide; LVEDV, LV end diastolic volume; LVESV, LV end systolic volume; SV, stroke volume; IVSd, interventricular septal diameter in diastole; LVPWd, LV posterior wall thickness in diastole; LVIDd, LV internal diameter in diastole.
