# Supplementary material for: The extracellular matrix proteoglycan fibromodulin is upregulated in clinical and experimental heart failure and affects cardiac remodeling
Source: PLoS One. 2018 Jul 27;13(7):e0201422. doi: 10.1371/journal.pone.0201422 (PMC6063439; doi:10.1371/journal.pone.0201422)
Supplement: S4 Table — (DOCX) [file pone.0201422.s012.docx]

**S4 Table. Characteristics of aortic-banded wild-type mouse cohort.**

|  | **Sham**  **24h** | **AB**  **24h** | **Sham**  **1w** | **AB**  **1w** | **Sham 3w** | **AB**  **3w** | **Sham 16w** | **AB**  **16w** | **Sham 18w** | **AB**  **18w** |
| --- | --- | --- | --- | --- | --- | --- | --- | --- | --- | --- |
| *Animal and organ weights* | | | | | | | | | | |
| N | 10 | 10 | 10 | 9 | 10 | 9 | 10 | 9 | 3-5 | 7-8 |
| Body weight (g) | 25.0 ±0.9 | 23.5  ±0.4 | 26.8  ±0.4 | 26.3  ±0.5 | 26.4  ±0.5 | 24.1  ±0.6* | 34.4  ±1.2 | 23.4  ±1.2*** | 36.4  ±2.4 | 25.9  ±1.9** |
| LVW/tibia (mg/mm) | 4.8  ±0.2 | 5.8  ±0.4* | 5.1  ±0.1 | 8.3  ±0.2*** | 5.2  ±0.2 | 8.9  ±0.2*** | 5.9  ±0.1 | 12.4  ±0.3*** | 5.9  ±0.4 | 12.2  ±0.2*** |
| LW/tibia (mg/mm) | 8.5  ±0.3 | 13.2  ±1.1*** | 8.9  ±0.2 | 20.0  ±1.4*** | 9.00  ±0.2 | 20.4  ±1.5*** | 9.4  ±0.1 | 23.9  ±1.3*** | 9.6  ±0.3 | 24.5  ±1.5*** |
| *Echocardiography (M-mode)* | | | | | | | | | | |
| N | 0 | 0 | 6 | 7 | 8 | 6 | 6 | 9 | 3 | 8 |
| LAD  (mm) | NA | NA | 1.70  ±0.02 | 3.02  ±0.09*** | 1.81  ±0.02 | 3.20  ±0.13*** | 1.70  ±0.09 | 3.49  ±0.20*** | 1.80  ±0.06 | 3.63  ±0.19*** |
| LVPWd (mm) | NA | NA | 0.75  ±0.03 | 1.08  ±0.02*** | 0.75  ±0.03 | 1.12  ±0.02*** | 0.82  ±0.05 | 1.08  ±0.02*** | 0.82  ±0.01 | 1.06  ±0.04** |
| LVIDd (mm) | NA | NA | 4.08  ±0.11 | 4.09  ±0.05 | 4.27  ±0.09 | 4.05  ±0.12* | 4.02  ±0.19 | 5.87  ±0.12*** | 4.26  ±0.05 | 5.75  ±0.14*** |
| FS (%) | NA | NA | 18.9  ±1.3 | 14.3  ±1.2* | 20.9  ±0.9 | 15.2  ±1.7* | 18.1  ±1.7 | 6.8  ±0.8*** | 15.3  ±1.6 | 6.7  ±0.9*** |
| *LV mRNA expression /RPL32* | | | | | | | | | | |
| N | 10 | 10 | 10 | 9 | 10 | 9 | 10 | 9 | 3 | 7 |
| NPPA | 1.00 ±0.52 | 3.19  ±0.73* | 1.00  ±0.10 | 10.99 ±1.65*** | 1.00  ±0.52 | 9.83 ±0.74*** | 1.00 ±0.14 | 48.35 ±4.89*** | 1.00  ±0.22 | 78.74 ±16.27* |
| NPPB | 1.00  ±0.09 | 11.46  ±1.52*** | 1.00  ±0.08 | 2.75  ±0.50** | 1.00  ±0.09 | 2.28  ±0.24*** | 1.00  ±0.13 | 6.21  ±0.18*** | 1.00  ±0.47 | 8.52  ±0.44*** |
| COL1A2 | 1.00  ±0.13 | 3.41  ±1.42*** | 1.00  ±0.07 | 15.35  ±1.83*** | 1.00  ±0.04 | 3.80  ±0.39** | 1.00  ±0.04 | 2.28  ±0.10*** | 1.00  ±0.10 | 3.23  ±0.33** |
| COL3A1 | 1.00  ±0.15 | 3.89  ±1.56*** | 1.00  ±0.08 | 13.10  ±1.63*** | 1.00  ±0.05 | 3.72  ±0.28* | 1.00  ±0.07 | 1.91  ±0.09*** | 1.00  ±0.13 | 2.41  ±0.27* |

Post-mortem and echocardiographic data (mean±SEM) of C57BL/6J wild-type (WT) mice 24 hours (h), 1-, 3-, 16-, and 18 weeks (w) after aortic banding (AB), and of sham-operated controls. N, number of animals; LVW, left ventricular weight; LW, lung weight; LAD, left atrial diameter; LVPWd, left ventricular posterior wall thickness in diastole; LVIDd, left ventricular internal diameter in diastole; FS, fractional shortening; NA, not available. Relative mRNA expression of the heart failure signature molecules NPPA and NPPB, and collagens I and III (COL1A2 and COL3A1), normalized to ribosomal protein L32 (RPL32) expression. Statistical differences were tested using an unpaired t-test vs. respective sham controls, *p<0.05; **p<0.01; ***p<0.001.
