# Supplementary material for: The extracellular matrix proteoglycan fibromodulin is upregulated in clinical and experimental heart failure and affects cardiac remodeling
Source: PLoS One. 2018 Jul 27;13(7):e0201422. doi: 10.1371/journal.pone.0201422 (PMC6063439; doi:10.1371/journal.pone.0201422)
Supplement: S5 Table — (DOCX) [file pone.0201422.s013.docx]

**S5 Table. Baseline characteristics of fibromodulin knock-out (FMOD-KO) and wild-type (WT) controls in adult, untreated mice.**

|  | **WT** | **FMOD-KO** |
| --- | --- | --- |
| *Animal and organ weights* | | |
| N | 8 | 8 |
| Body weight (g) | 24.0±0.6 | 25.4±1.0 |
| HW/BW (mg/g) | 4.8±0.2 | 4.9±0.1 |
| LW/BW (mg/g) | 5.6±0.1 | 5.9±0.1 |
| Tibia (mm) | 17.0±0.1 | 17.6±0.2* |
| *Blood pressure* | | |
| N | 12 | 12 |
| Systolic BP (mm/Hg) | 108±2 | 109±4 |
| Diastolic BP (mm/Hg) | 85±3 | 85±4 |
| *Echocardiography (M-mode)* | | |
| N | 10 | 10 |
| IVSd (mm) | 0.72±0.02 | 0.75±0.02 |
| LVPWd (mm) | 0.71±0.02 | 0.74±0.01 |
| LVIDd (mm) | 4.31±0.06 | 4.15±0.10 |
| LAD (mm) | 1.81±0.03 | 1.82±0.03 |
| FS (%) | 19.5±1.0 | 21.2±1.4 |
| *LV mRNA expression /RPL32* | | |
| N | 8 | 8 |
| NPPA | 1.00±0.16 | 0.91±0.07 |
| NPPB | 1.00±0.17 | 0.72±0.24 |

Post-mortem and echocardiographic data (mean±SEM) of adult, untreated FMOD-KO and WT control mice (C57BL/6N) at baseline (7-9 weeks of age, n=8-12). HW, heart weight; BW, body weight; LW, lung weight; BP, blood pressure; IVSd, interventricular septum thickness in diastole; LVPWd, left ventricular posterior wall thickness in diastole; LVIDd, left ventricular internal diameter in diastole; LAD, left atrial diameter; FS, fractional shortening; Relative mRNA expression of heart failure signature molecules NPPA and NPPB, normalized to ribosomal protein L32 (RPL32) expression. Statistical differences were tested using an unpaired t-test vs. WT, *p≤0.05.
